# The *fast-food effect*: costs of being a generalist in a human-dominated landscape

**DOI:** 10.1093/conphys/coad055

**Published:** 2023-08-14

**Authors:** Sergio Guerrero-Sanchez, Liesbeth Frias, Silvester Saimin, Pablo Orozco-terWengel, Benoit Goossens

**Affiliations:** Centre for Applied One Health Research and Policy Advice, Jockey Club College of Veterinary Medicine and Life Sciences, City University of Hong Kong. To Yuen Building. 31 To Yuen Street, Kowloon, HK; Organisms and Environment Division, School of Biosciences, Cardiff University, Sir Martin Evans Building, Museum Avenue, Cardiff CF10 3AX, UK; Duke-NUS Medical School, Programme in Emerging Infectious Diseases. No. 8 College Road, Singapore 169857; Asian School of the Environment, Nanyang Technological University, 50 Nanyang Avenue, Singapore 639798; Sabah Wildlife Department, 5th Floor, Block B, Wisma Muis, Jalan Access Bomba Negeri, Kota Kinabalu, Sabah, 88100 Malaysia; Organisms and Environment Division, School of Biosciences, Cardiff University, Sir Martin Evans Building, Museum Avenue, Cardiff CF10 3AX, UK; Organisms and Environment Division, School of Biosciences, Cardiff University, Sir Martin Evans Building, Museum Avenue, Cardiff CF10 3AX, UK; Sabah Wildlife Department, 5th Floor, Block B, Wisma Muis, Jalan Access Bomba Negeri, Kota Kinabalu, Sabah, 88100 Malaysia; Danau Girang Field Centre, c/o Sabah Wildlife Department, 5th Floor, Block B, Wisma Muis, Jalan Access Bomba Negeri, Kota Kinabalu, Sabah, 88100 Malaysia

**Keywords:** animal health, Asian water monitor lizard, blood chemistry, Borneo, diet, oil palm, parasites

## Abstract

Agricultural expansion in Southeast Asia has converted most natural landscapes into mosaics of forest interspersed with plantations, dominated by the presence of generalist species that benefit from resource predictability. Dietary shifts, however, can result in metabolic alterations and the exposure of new parasites that can impact animal fitness and population survival. Our study focuses on the Asian water monitor lizard (*Varanus salvator*), one of the largest predators in the Asian wetlands, as a model species to understand the health consequences of living in a human-dominated landscape in Sabah, Malaysian Borneo. We evaluated the effects of dietary diversity on the metabolism of monitor lizards and the impact on the composition of their parasite communities in an oil palm-dominated landscape. Our results showed that (1) rodent-dominated diets were associated with high levels of lipids, proteins and electrolytes, akin to a *fast-food*-based diet of little representativeness of the full nutritional requirements, but highly available, and (2) lizards feeding on diverse diets hosted more diverse parasite communities, however, at overall lower parasite prevalence. Furthermore, we observed that the effect of diet on lipid concentration differed depending on the size of individual home ranges, suggesting that sedentarism plays an important role in the accumulation of cholesterol and triglycerides. Parasite communities were also affected by a homogeneous dietary behaviour, as well as by habitat type. Dietary diversity had a negative effect on both parasite richness and prevalence in plantations, but not in forested areas. Our study indicates that human-dominated landscapes can pose a negative effect on generalist species and hints to the unforeseen health consequences for more vulnerable taxa using the same landscapes. Thus, it highlights the potential role of such a widely distributed generalist as model species to monitor physiological effects in the ecosystem in an oil palm-dominated landscape.

## Teaser

In this study, we use *Varanus salvator* as a model species to present the physiological implications of living in a highly anthropogenic landscape, as well as the consequences on the parasite community associated with dietary shifts.

## Introduction

Agricultural expansion in the tropics has converted most natural landscapes into mosaics of forest and industrialized crops, altering the availability and distribution of food resources for a wide range of wildlife (Oro *et al.,* 2013). While for some, taxa deforestation and land use change can result in resource depletion, for others, anthropogenic changes in the landscapes offer an abundant food source with human-base resources that are both abundant and predictable (McKinney, 2006; Newsome *et al.,* 2014). As a result, many wildlife species have adapted to benefit from these resources, leading to larger populations that are more aggregated and better fed (Prange *et al.,* 2003). Raccoons (*Procyon lotor*), for example, have been reported to have higher population densities and survival rates in urban and suburban areas in Northern United States and Canada, as well as very low weight loss during winter, compared to those living in more natural environments (Hoffmann, 1979; Rosatte *et al.,* 1991; Prange *et al.,* 2003). Moreover, shifts in intra- and interspecies interactions are more likely to be observed in human-modified landscapes (Ciucci *et al.,* 2020), including an increased risk of human–wildlife contact (Hopkins III *et al.,* 2014), favouring changes in pathogen host range and distribution (Patz *et al.,* 2000; Gillespie and Chapman, 2006; Lafferty *et al.,* 2006; Cardoso *et al.,* 2016; Bonnell *et al.,* 2018).

In Asia, one of the largest beneficiaries of anthropogenic food is the Asian water monitor lizard (*Varanus salvator*), one of the largest predators in the region’s wetlands (Traeholt, 1994; Guerrero-Sanchez *et al.,* 2021). The species natural habitat is linked to lowland freshwater, such as mangroves, swamps and wetlands under 1000 m above the sea level (m.a.s.l.; Horn and Gaulke, 2004), where they feed on a large range of animals, such as fish, frogs, invertebrates, birds, small mammals and animal carcasses (Traeholt, 1994; Uyeda, 2009). Nonetheless, monitor lizards are exceptionally persistent in anthropogenic habitats, being particularly abundant in the proximity of farms, households and urban areas (Uyeda, 2009; Karunarathna *et al.,* 2017). High food availability, as well as reduced areas of suitable habitat for the species, has driven lizards inhabiting oil palm habitats to a sedentary behaviour, establishing home ranges that are significantly smaller than those of lizards living in natural forest (Guerrero-Sanchez *et al.,* 2022). Consequently, these adaptations to agricultural landscapes have induced changes in their ecology and behaviour, altering their physiology through a higher accumulation of blood metabolites and exposure to novel parasite assemblages (Jessop *et al.,* 2012; Smyth *et al.,* 2014).

Dietary shifts induced by human-modified habitats have physiological consequences for wildlife health, as they often provide resources that may vary considerably in terms of quality, energy and nutrient composition from natural diets (Oro *et al.,* 2013). For example, body condition can increase as a result of a higher and predictable intake of calories (Kaneko and Maruyama, 2005), or decrease if anthropogenic food is low quality (Liker *et al.,* 2008). Anthropogenic food can boost wildlife body condition and increase immune defences, as shown in lace monitors (*Varanus varius*) foraging on human subsidies in Australia, where not only provisioned individuals were larger and heavier than non-provisioned animals, but they also showed lower intensity of blood parasites (Jessop *et al.,* 2012). By being localized, anthropogenic food can also reduce wildlife movement and foraging time (Murray *et al.,* 2015), which could be compared to the accessible and affordable fast food for humans. Just like fast food has consequences to human health (Davis and Carpenter, 2009), shifts to a more homogeneous and highly caloric diet also have physiological implications in animals. Lace monitors feeding on human subsidies have reported higher levels of creatinine kinase and aspartate aminotransferase compared to those feeding on more natural diets (Jessop *et al.,* 2012).

Parasite diversity, on the other hand, is strongly linked to the host dietary behaviour (Lafferty, 1999; Poulin and Morand, 2004). Thielges and Poulin (2016), for example, found that the prey range in birds at the Pacific Coast of the United States and Mexico is positively associated with parasite richness, where birds with broader diets showed a higher parasite richness. Host susceptibility to infection and higher parasite loads can also be the consequence of a poor-quality diet (Ezenwa, 2004). For example, rock iguanas (*Cyclura cychlura*) supplementary fed with processed food, rich in carbohydrates and sugar, not only presented an altered nutritional status but also showed increased prevalence of hookworm (Knapp *et al.,* 2013). In other species, the induced sedentary behaviour, reflected in the reduction of the individual home range and population aggregation, increases exposure to infected conspecifics, as well as the shedding and accumulation of pathogens into the environment (Becker and Hall, 2014; Gilbert *et al.,* 2016).

Here, we evaluated the influence of dietary diversity on the physiological responses and exposure to parasites of Asian water monitor lizards in an oil palm-dominated landscape. By providing reliable food resources, oil palm (*Elaeis guineensis*) plantations could boost the body condition of lizards, increase their immune defences and reduce their foraging time, which in turn could reduce parasite fitness by decreasing individual susceptibility to infection and promoting quick recovery (Becker *et al.,* 2015). We hypothesized that the feeding behaviour of plantation lizards was less diverse than that of lizards living in the forest, and that this tendency would be reflected in (1) higher values of biochemical markers associated with shifts in the dietary diversity and (2) a reduction in overall parasite diversity and increased parasite prevalence. Using *V. salvator* as model species, we aim to contribute to the understanding of the physiological implications of species adaptations to human-dominated landscapes and provide information that can be extrapolated to other more cryptic and vulnerable species.

## Materials and Methods

### Study area

**Figure 1:**
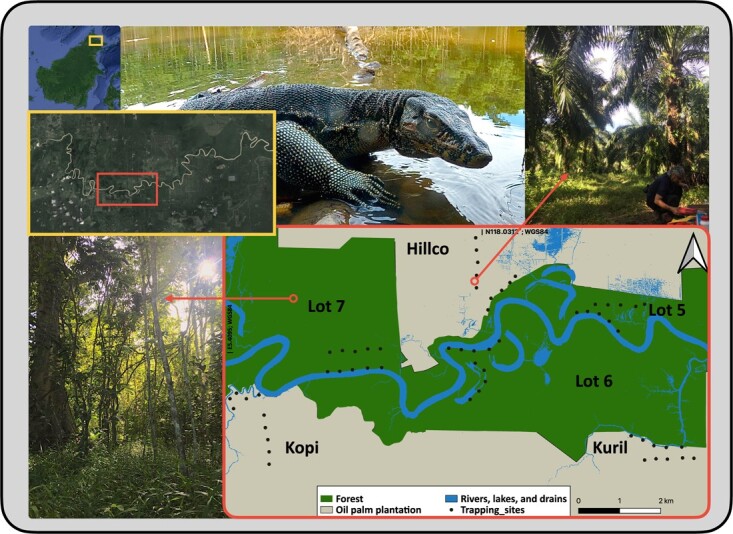
Study area in Malaysian Borneo (top left corner). Trapping sites (bottom right map) were distributed across three forest lots and three oil palm estates within the LKWS (upper left maps). Images are representative of both forest (bottom left) and OPP (top right) sites. The lizard in the top centre is one of the sampled individuals weighting ~ 20 kg. Photos ©L. Frias (upper right and bottom left) and ©R. Delvaux (Monitor lizard). Source of landscape images: Google Earth (2019).

The study was conducted in the Kinabatangan floodplain (5°10′–5°50′N; 117°40′–118°30′E), located in the east coast of Sabah (Malaysian Borneo; Figure 1). The floodplain (~30 m.a.s.l.) consists of a complex matrix of different types of forest interspersed with rural settlements and large extensions of oil palm crops along the Kinabatangan River, which, along with oxbow lakes and tributaries, irrigate the landscape either seasonally or permanently (Estes *et al.,* 2012). Along the Kinabatangan River, the Lower Kinabatangan Wildlife Sanctuary (LKWS) expands in a series of patches of protected forest (hereafter referred to as “lots”) encroached between the main river and industrial palm oil. Connectivity among these lots, and between lots and other forested areas, is generally poor, often consisting in narrow strips of highly degraded forest, and in the worst cases, completely absent (Ancrenaz *et al.,* 2004; Abram *et al.,* 2014).

### Animal sampling

We spent a total of 3055 traps/day between October 2013 and September 2016, split in three forest lots (Lot 5, 6 and 7) within the LKWS and three surrounding oil palm plantation (OPP) estates (Hillco, Kopi and Kuril; Figure 1). We established two transects in each site and placed one cage trap every 400 m for a total of five traps per transect (Guerrero-Sanchez *et al.,* 2021). Traps were then opened and baited in the early morning, using chicken entrails, and checked in the early afternoon. Asian water monitor lizards were grouped by habitat (forest/plantation) and site (forest lot/plantation estate), according to where they were trapped. Since monitor lizards, especially those living in the boundaries, can roam in both types of habitats, using OPP as food sources and forested areas as a shelter (Guerrero-Sanchez *et al.,* 2021), we established a third group for those that were captured in both types of habitats to avoid bias or pseudo-replications. All trapped lizards were tagged with an intradermal transponder (ID-1AA; Trovan LTD, UK).

Each lizard was weighted, and body length was measured from the tip of the mouth to the cloaca (snout to vent length). We also collected up to 2 ml of blood from the coccygeal vein for the biochemical analysis, which included lipids, proteins and electrolytes. Although it is safe to collect up to 5% of blood in relation to body weight (Jacobson, 1993), to minimize the risk of lesions caused by the venepuncture (i.e. accidental pinch of the caudal nerves or rupture of the vein), we only collected blood from individuals over 3 kg. Due to difficulties in sex determination by physical methods, not all individuals were sexed unequivocally. Frynta *et al.* (2010) reported that male varanids are three times larger than females. Therefore, by being unable to determine the sex of all individuals, we could not estimate their age range, and we omitted sex and age as independent variables in the analysis.

### Diet inventory

As a proxy to describing the dietary diversity of monitor lizards in the study site, we recorded an inventory of stomach contents of all trapped lizards. Regurgitation is a common response mechanism to threatening situations (e.g. being trapped) in reptiles (Greene, 1988), and thus, it was anticipated to happen in most (if not all) of the trapped individuals during the study. The regurgitated content was collected, and food items were identified and classified into taxonomic groups, according to morphological features. The food items recorded in this study only represent what captured individuals ate prior to being captured and are likely informative of local food availability rather than dietary preferences. We used the term “dietary diversity” instead of “prey diversity” to avoid any confusion with individual preferences.

### Biochemical analysis

Blood samples were processed within 2 hours of collection, and the serum was separated from blood cells through centrifugation (1000 × *g* for 10 min) and kept at −20°C until analysis (Fudge, 2000). Samples were analysed for a full biochemistry panel in a commercial laboratory (Gribbles Sdn. Bhd., Sandakan, Malaysia). For this study, we selected a set of biochemical elements (hereafter *biomarkers*) commonly associated with dietary behaviour. Cholesterol (including low- and high-density lipoproteins [LDL-Ch and HDL-Ch, respectively]) and triglycerides are indicators of energy intake, use and storage in an individual. They are also commonly associated with highly caloric food and, together with sedentary behaviour, drivers of cardiovascular diseases (Meyer and Harvey, 2004). Proteins are a significant part of an individual’s metabolism, as they are fundamental for body structure and functionality. Low-quality proteins may be reflected in the ability of an animal to perform its biological functions and make it more vulnerable to both metabolic and infectious diseases (Meyer and Harvey, 2004). Uric acid serves as an indicator of protein metabolism, usually of concern when there is an exceedingly high intake of proteins (Meyer and Harvey, 2004). Electrolytes, such as sodium, potassium and chloride, are essential in tissue homeostasis and strongly related to muscular functionality (Meyer and Harvey, 2004). They are also associated with a high consumption of processed food, as they are a main component of artificial flavouring and preservatives (Meyer and Harvey, 2004).

### Parasite sampling and identification

A cleaned plastic tarp was placed under each trap to collect faecal samples and avoid contamination with free-living nematodes. All faecal samples were processed using a modified formalin-ethyl acetate sedimentation protocol to concentrate parasite eggs, and then samples were examined with a sequential sedimentation–flotation procedure (Frias *et al.,* 2021). We evaluated two measures of parasite infection influenced by dietary diversity: (i) parasite species richness, calculated as the number of parasite taxonomic groups identified in an individual, and (ii) parasite prevalence, expressed as the percentage of individuals positive for a given parasite taxonomic group (Bush *et al.,* 1997).

### Statistical analysis

Dietary diversity was estimated for each sampling site using the Shannon–Wiener diversity Index (H′). Differences between sites and habitats were calculated by analysis of their variance. Body condition (BC), i.e. an index that reflects the physiological and nutritional status of an individual (Labocha *et al.,* 2014), was estimated as the linear regression between log-transformed body weight and log-transformed body length (Green, 2001).

Differences for each biomarker were assessed with generalized linear models (GLM) using two different categorical variables, i.e. habitat (forest vs. OPP) and sites (3 forest lots and 3 oil palm estates). Later, we grouped the study sites according to the habitat type and evaluated the differences within each group. Each biomarker GLM was set with the corresponding family distribution error. GLM were carried out with the stats v3.6.3 package for R (R Core Team) and validated using the diagnostics for hierarchical regression models (DHARMa, v.0.4.6; Hartig, 2022). Distribution for both biochemical markers and parasite data was assessed using the fitdistrplus v1.1.8 package for R (R Core Team; Delignette-Muller and Dutang, 2015) and is presented in Section 2 of the supplementary material (fit of distribution for the biochemical values, BC, and parasite data by maximum likelihood estimation).

To determine if dietary diversity accounted for a significant proportion of the variation between habitats and among sites for each of the biomarkers assessed, generalized estimation equations (GEE) were run by using the geeglm function in geepack v.1.3-1 (Halekoh *et al.,* 2006). Contrary to other GLM that estimate a within-group variance component, GEE models estimate the average response of each group, considering the most suitable between-group correlation structures (Hardin and Hilbe, 2002; Yan and Fine, 2004; Zuur *et al.,* 2009). Correlation structures were set either as exchangeable or unstructured, while the distribution family error was defined properly for each marker’s distribution. All the GEE models in this study were validated with the Pearson correlation test of the residuals.

Since sedentarism is a complementary variable to dietary behaviour in the metabolism of energy, we use lizards’ home range as a proxy to activity pattern. A subset of 10 individuals between 15 and 20 kg (presumed adult males) was extracted from the whole sampled population to understand the effect of home range size over the association between dietary diversity and the value of the biochemical markers. Those lizards belonged to a group of 14 individuals tagged with GPS trackers, and which home ranges were defined in a previous study using local convex hull (Guerrero-Sanchez *et al.,* 2022), and all of them were tested for total cholesterol, nine for proteins and electrolytes and eight for triglycerides and LDL-Ch and HDL-Ch . In this case, GEE models were set with either an independent or exchangeable correlation structure, and like in the analysis mentioned above, a proper distribution error was set for each biomarker. Here, the models were also validated with the Pearson correlation of the residuals.

Parasite richness and prevalence were estimated per site and habitat type, although only prevalence was compared per habitat type and per site using GLM with Poisson distribution errors. Prevalence among habitats and sites was also compared per each one of the parasitic groups with major presence in the study. The effect of dietary diversity and BC on both parasite richness and prevalence was evaluated with GEE models. In the case of prevalence, models were performed with the overall parasite community and, finally, for each one of the parasitic groups with major presence in the study. All models were set with a Poisson error distribution and exchangeable autocorrelation structure. Models were evaluated using the Pearson test of the residuals.

## Results

We captured and marked 402 unique individuals during the study period (3055 traps/day). Capture data showed that individuals were recaptured within the same transect as in the first time, and none of the individuals were caught in both OPP and forest. Thus, lizards were grouped only in two categories (forest and OPP). Data on blood metabolites were available for 256 individuals, and only 73 faecal samples were collected and suitable for the study.

### Diet

**Figure 2:**
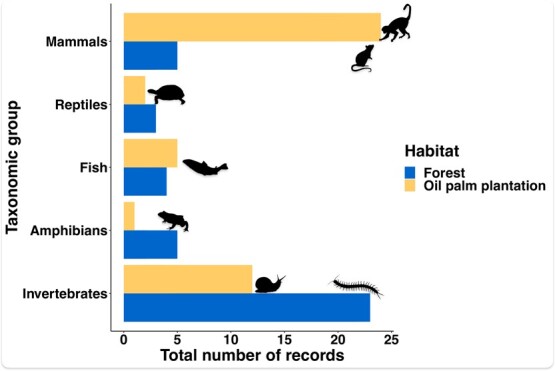
Diet inventory identified in the vomit of Asian water monitor lizards.

The dietary inventory was established based on the stomach content of 132 individuals, and it included 182 prey items categorized into 14 taxonomic groups. The remaining 124 individuals were discarded since they did not vomit or only vomited the bait. Overall, dietary diversity was significantly higher in forest lots than in OPP (H′_Forest_ = 2.114 vs. H′_Plantation_ = 1.052; *F* = 9309; *P* < 0.001). Invertebrates such as crabs, centipedes and woodlice made up an important part of the diet of lizards in forested areas (54% of the records), while rodents were the dominant prey in plantations (Table 1 and Figure 2). Fish were almost exclusively represented by a catfish species from the genus *Pterygoplichthys,* invasive to Borneo and Southeast Asia.

**Table 1:** Dietary diversity, presented as Shannon–Wiener index (H′), calculated for prey items identified in the vomit of Asian water monitor lizards

Prey type	Forest			OPP		
	Lot 5	Lot 6	Lot 7	Hillco	Kopi	Kuril
Arthropods						
Centipede	4	1	3	2	1	3
Crab	10	6	5	0	0	0
Scorpion	1	0	1	0	0	0
Woodlouse	4	3	10	2	2	8
Amphibians						
Frog	5	1	2	0	1	0
Fish						
Catfish	0	4	1	0	2	5
Reptiles						
Egg (snake)	1	1	0	0	0	0
Snake	1	1	0	0	1	0
Tortoise	1	1	1	0	1	0
Mollusca						
Snail	3	3	4	0	2	1
Mammals						
Bat	0	1	0	0	0	0
Macaque	1	0	1	0	0	0
Rodent	3	2	2	24	20	18
Wild boar	1	0	0	0	0	0
H′	2.162	2.167	1.978	0.509	1.265	1.269

**Table 2:** Significant outcomes of the statistical analysis carried out for the biochemical markers and parasite prevalence and richness

Statistic	Dependent variable	Independent variable	Outcome
Biochemical markers			
GLM	Total cholesterol	Forest vs. oil palm	*X^2^* = 2.80, *P* = 0.03
	Total proteins	Forest vs. oil palm	*X^2^* = 446.66, *P* = 0.01
	Globulin	Forest vs. oil palm	*X^2^* = 0.10, *P* = 0.02
	Total cholesterol	All sites	*X^2^* = 18.36, *P* = 0.03
	LDL-cholesterol	All sites	*X^2^* = 9.16, *P* < 0.01
	HDL-cholesterol	All sites	*X^2^* = 2.61, *P* < 0.01
	Total proteins	All sites	*X^2^* = 898.17, *P* = 0.03
	Albumin	All sites	*X^2^* = 136.53, *P* = 0.05
	HDL-cholesterol	Forest sites	*X^2^* = 2.01, *P* = 0.01
	Total cholesterol	Oil palm sites	*X^2^* = 14.20, *P* < 0.01
	LDL-cholesterol	Oil palm sites	*X^2^* = 7.18, *P* < 0.01
	Total proteins	Oil palm sites	*X^2^* = 390.84, *P* = 0.04
	Albumin	Oil palm sites	*X^2^* = 113.59, *P* = 0.01
GEE	LDL-cholesterol	Dietary diversity (F)	*β* = −0.37 (0.16), *P* = 0.038
	LDL-cholesterol	Dietary diversity (OPP)	*β* = −0.80 (0.33), *P* = 0.016
	HDL-Cholesterol	Dietary diversity (F)	*β* = −0.12 (0.05), *P* = 0.022
	HDL-Cholesterol	Dietary diversity (OPP)	*β* = −0.20 (0.08), *P* = 0.009
	Triglycerides	Dietary diversity (F)	*β* = 0.07 (0.28), *P* = 0.81
	Triglycerides	Dietary diversity (OPP)	*β* = −1.46 (0.47), *P* = 0.002
	Total protein	Dietary diversity (F)	*β* = 5.33 (2.32), *P* = 0.021
	Total protein	Dietary diversity (OPP)	*β* = 21.60 (5.82), *P* < 0.001
	Potassium	Dietary diversity (F)	*β* = −2.23 (0.19), *P* = 0.061
	Potassium	Dietary diversity (OPP)	*β* = −5.25 (1.98), *P* = 0.008
	BC	Dietary diversity + HR	*β* = 0.33 (0.11), *P* = 0.003
	LDL-cholesterol	Dietary diversity + HR	*β* = 1.24 (0.33), *P* < 0.001
	HDL-cholesterol	Dietary diversity + HR	*β* = 0.24 (0.12), *P* = 0.043
	Triglycerides	Dietary diversity + HR	*β* = 1.13 (0.68), *P* = 0.002
Parasites			
GLM	Prevalence	Forest vs. oil palm	*X^2^* = 125.66, *P* < 0.001
	Prevalence	All sites	*X^2^* = 87258.21, *P* < 0.001
	*Trichuris* prev.	Forest vs. oil palm	*X^2^* = 2075.20, *P* < 0.001
	Spirurids	Forest vs. oil palm	*X^2^* = 864.32, *P* < 0.001
	Oxyurids	Forest vs. oil palm	*X^2^* = 3771.04, *P* = 0.002
	*Strongyloides*	Forest vs. oil palm	*X^2^* = 1605.39, *P* < 0.001

**Table 2:** Continued

Statistic	Dependent variable	Independent variable	Outcome
GEE	Parasite richness	BC (F)	*β* = −0.45 (1.521), *P* = 0.77
	Parasite richness	BC (OPP)	*β* = 2.23 (0.739), *P* = 0.002
	Trichuris prevalence	Dietary diversity	*β* = 0.59 (0.27), *P* = 0.03
	Physaloptera prevalence	Dietary diversity	*β* = −0.665 ± 0.25, *P* = 0.008
	*Strongyloides* prevalence	Dietary diversity	*β* = −0.374 ± 0.19, *P* = 0.058
	Parasite richness	Dietary diversity (F)	*β* = 9.37 (1.62), *P* < 0.001
	Parasite richness	Dietary diversity (OPP)	*β* = −22.50 (0.36), *P* < 0.001
	Parasite prevalence	Dietary diversity (F)	*β* = 32.8 (12.1), *P* = 0.007
	Parasite prevalence	Dietary diversity (OPP)	*β* = −6.27 (2.44), *P* = 0.011

Here we only presented the significant results. The full outcome is presented in the supplementary material.

F indicates forest; OPP, oil palm plantation; HR, home range size.

### Body condition

Overall, lizards in OPP had a slightly higher BC than those living in the forest (BC_OPP_ = 0.389 ± 0.12; BC_Forest_ = 0.353 ± 0.15). Among forest lizards, those captured in Lot 5 (BC_Lot5_ = 0.391 ± 0.13), where the trapping site was placed less than 700 m from the plantation boundaries, had a slightly higher BC than those captured in sites placed farther than 700 m from the plantation boundaries (BC_Lot6_ = 0.337 ± 0.17; BC_Lot7_ = 0.345 ± 0.13). However, we did not find significant differences among habitats or among sites (Supplementary Tables 1 and 2).

### Biomarkers

Lizards inhabiting forested areas presented significantly higher levels of total cholesterol (Forest = 2.04 mmol/l vs. OPP = 1.8 mmol/l), while lizards in OPPs presented higher levels of total proteins (OPP = 80.3 g/l, Forest = 76.6 g/l) and globulin (OPP = 54.1 g/l, Forest = 51.1 g/l; Table 1). We also found significant differences among all the sites for total cholesterol, LDL-Ch, HDL-Ch, total proteins and albumin. However, when we compared sites grouped per type of habitat, only high-density cholesterol levels were different among forested sites, while the rest of the markers did not show significant variations. OPP sites, on the contrary, presented significant differences among them for total cholesterol, LDL-Ch, total proteins and albumin (Table 2, see also Supplementary Tables 1 and 2).

### The influence of habitat type and home range size

**Figure 3:**
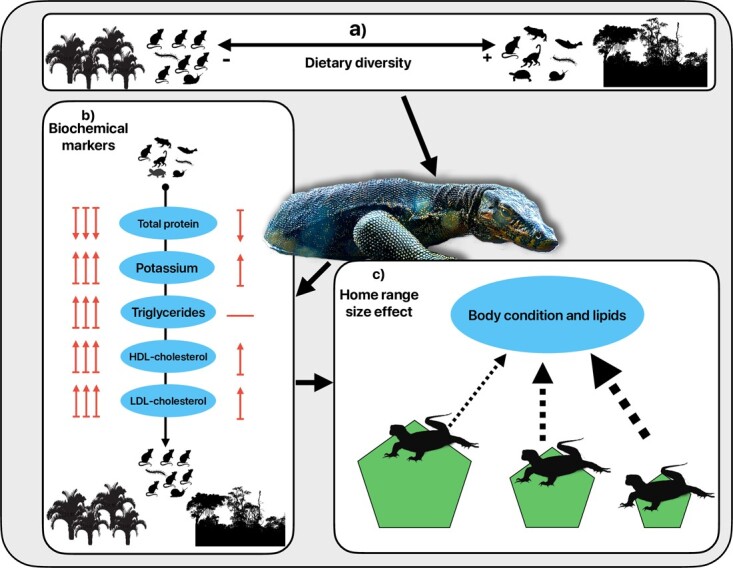
Graphic representation of the impact of dietary diversity on the biochemical markers of Asian water monitor lizards. Forest lizards have a more diverse prey availability, while those living in OPPs primarily feed on rodents (a). The impact of dietary diversity on certain biochemical markers varies in magnitude and direction based on the type of habitat (b). In addition, the effect of dietary diversity on BC and lipids is enhanced by the size of an individual’s home range, with smaller ranges intensifying the negative correlation between dietary diversity and these biomarkers (c). Lizard image edited from ©R. Delvaux. Animal and trees' shape source: Phylopic.org.

The results of the GEE models showed that dietary diversity had a significant negative effect on low- and high-density lipoprotein cholesterol in monitor lizards, but that this effect was stronger in plantation areas (Figure 3b and Table 2. See also Supplementary Table 3). Total proteins, on the other hand, increased with dietary diversity, and the association was stronger in OPP than in forested areas. Triglycerides and potassium showed significantly higher levels associated with low dietary diversity in OPP, but not in forested areas.

**Figure 4:**
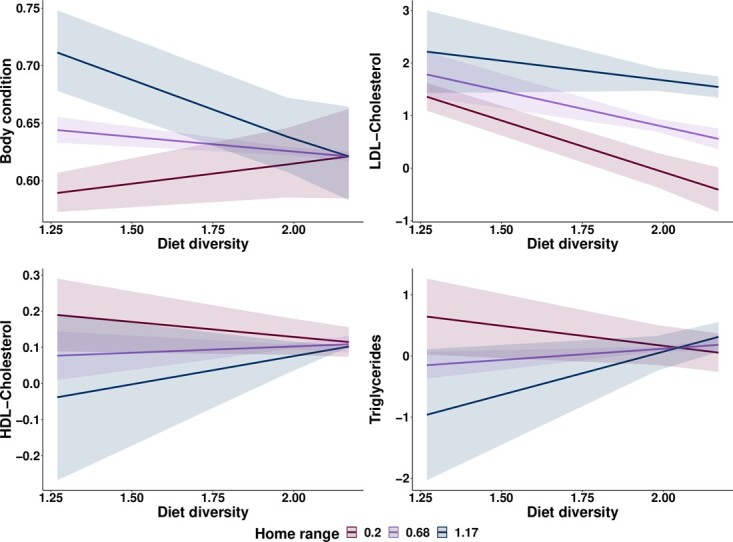
Predictive effects of dietary diversity on BC, LDL and HDL cholesterol, triglycerides, and their variation based on lizards’ home range sizes. Home range, estimated as local convex hull with adaptive algorithm, is expressed in kilometer squared.

For those samples with a known home range size, the intensity of the effect of dietary diversity on BC and three fat-related biomarkers showed to be dependent on home range size (Figure 3c and 4, see also Table 2 and Supplementary Table 4). BC decreased with higher dietary diversity in individuals with larger home ranges. However, when home ranges shrank, this correlation gradually shifted tendencies, with BC increasing along with dietary diversity. Reduction of home range size also boosted a negative association between low-density cholesterol dietary diversity. HDL cholesterol and triglycerides were higher in individuals with smaller home ranges and lower dietary diversity, but the intensity and direction of the effect shifted gradually from negative to positive with increasing home range sizes.

### Parasite species richness and prevalence

Faecal samples were collected from 73 lizards (*n*_Forest_ = 32; *n*_Plantation_ = 41), and overall parasite prevalence was estimated at 79.4% (*n* = 58). During our trapping period, conditions surrounding the Kuril estate were inadequate for faecal sample collection, i.e. there was an excess of mud due to continuous rain and flooding, and thus, data from this study site were not included in the analysis. A total of 10 parasite taxonomic groups were identified, corresponding to nematodes (*n* = 8), cestodes (*n* = 1) and trematodes (*n* = 1; Table 3). Nematodes included parasites from the genus *Capillaria* (42.4%), *Strongyloides* (17.8%), *Trichuris* (9.5%), *Physaloptera* (5.4%) and *Ascaris* (1.3%), and from the orders Oxyurida (35.6%), Strongylida (26%) and Spirurida (5.4%).

**Table 3:** Parasites reported from lizards’ faeces

Parasite taxonomic group	Lot 5 (*n* = 12)	Lot 6 (*n* = 5)	Lot 7 (*n* = 14)	Forest (*n* = 31)	Hillco (*n* = 12)	Kopi (*n* = 30)	Oil palm (*n* = 41)
Nematodes							
*Ascaris* spp.	0	0	7.1 (1)	3.1 (1)	0	0	0
*Capillaria* spp.	53.9 (7)	0	35.7 (5)	37.5 (12)	41.7 (5)	48.3 (14)	46.3 (19)
Oxyurida spp.	53.9 (7)	0	50 (7)	43.8 (14)	58.3 (7)	17.2 (5)	29.3 (12)
*Physaloptera* spp.	7.7 (1)	20 (1)	0	6.2 (2)	16.7 (2)	0	4.9 (2)
Spirurida spp.	7.7 (1)	20 (1)	7.1 (1)	9.4 (3)	8.3 (1)	0	2.4 (1)
Strongylida spp.	15.4 (2)	80 (4)	7.1 (1)	21.9 (7)	16.7 (2)	34.5 (10)	29.3 (12)
*Strongyloides* spp.	30.8 (4)	0	0	12.5 (4)	25 (3)	20.7 (6)	22 (9)
*Trichuris* spp.	7.7 (1)	40 (2)	14.3 (2)	15.6 (5)	0	6.9 (2)	4.9 (2)
Cestodes							
Cestoda	0	0	7.1 (1)	12.5 (1)	8.3 (1)	0	2.4 (1)
Trematodes							
Trematoda	0	60 (3)	0	9.4 (3)	0	0	0
Overall prevalence	76.9 (10)	80 (4)	71.4 (10)	74.9 (24)	83.3 (10)	80 (24)	80.9 (34)
Parasite species richness	7	5	7	10	7	5	8

Values show prevalence (%) by site, followed by the number of positive individuals for each parasite. Overall prevalence per site and habitat type, as well as parasite richness, are included in the lower rows of the table

Out of the 10 taxonomic groups of helminths identified, all of them were found in forest, while only eight were detected in OPPs, where *Ascaris* spp. and Trematoda were absent. Parasite prevalence, on the other hand, was significantly higher in plantation estates than in forest sites (Table 2). We also found significant differences among sites, where the Hillco estate showed the highest parasite prevalence (83%) among the study sites, followed by the Kopi estate and Lot 5 (80%) and Lot 6 (71.4%). Forest lizards showed significantly higher prevalence of *Trichuris* spp. (15.6% vs. 4.8% in OPP), spirurids (9.37% vs. 2.44%) and oxyurids (43.8% vs. 29.3%), while plantation lizards had significantly higher prevalence of parasites of the genus *Strongyloides* spp. (22% vs. 12.5%). Cestodes were only found in two individuals, one in Lot 7 and in one in the Hillco estate, while parasites of the genus *Ascaris* spp. were found only in Lot 7. Trematodes, on the hand, were only found in three individuals in Lot 6.

Positive association between parasite richness and BC was observed in OPP sites, but not in forested areas (Table 2). Association between BC and prevalence was found neither in forest nor OPP Supplementary Table 5). For specific parasites, the prevalence of *Trichuris* spp. was higher in areas of high dietary diversity, while the prevalence of *Physaloptera* spp. and *Strongyloides* spp. was higher in areas with less diverse diet (Table 2 and Supplementary Table 5).

**Figure 5:**
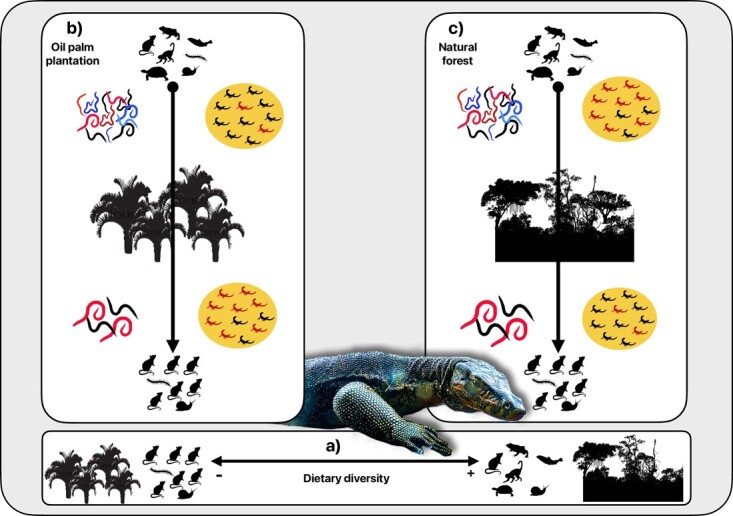
Graphic representation of the impact of dietary diversity on parasite communities associated with the Asian water monitor lizard. OPPs are associated with a decrease in parasite species richness and higher parasite prevalence (a). In contrast, in forested areas, an increase in dietary diversity leads to an increase in both the number of parasite taxonomic groups (richness) and parasite prevalence (b). Lizard image edited ©R. Delvaux. Animal and trees' shape source: Phylopic.org.

Regarding the effect of habitat on the parasite community associated with the monitor lizards, we found that dietary diversity had a significant effect on both parasite richness and prevalence, and this effect is different according to the type of habitat. While in the forest, sites with more heterogeneous diet shows a positive tendency in such associations; they become negative in OPP (Figure 5 and Supplementary Table 5).

## Discussion

As part of a broader study on the ecology of the Asian water monitor lizard and the effect of a changing landscape on its populations in Borneo (Guerrero-Sanchez *et al.,* 2021, 2022), this research investigated one of the consequences of being a generalist carnivore in a human-dominated landscape. There is a wealth of information available on the species in the study area, including its population dynamics and habitat use. Combined with the findings of the current study, this information presents an opportunity to develop a model species that might help us understand the physiological implications of oil palm for animal communities in Borneo.

### Dietary diversity

Environmental changes that drive animal populations to dietary shifts have a substantial impact on animal physiology and can result in cascading effects across interaction networks, including host–parasite interactions (Prange *et al.*, 2003; Ezenwa, 2004; Kaneko and Maruyama, 2005; Liker *et al.,* 2008). In Southeast Asia, large areas of forest have been converted to extensive agriculture, providing Asian water monitor lizards, and other mesopredators, with habitats where foraging efforts are considerably lower than in natural habitats, and where rewards, represented by abundant sources of animal protein and human subsidies, are significantly higher (Becker *et al.,* 2015; Jennings *et al.,* 2015; Guerrero-Sanchez *et al.,* 2022). Our study shows that oil palm plantations provide monitor lizards with a lower dietary diversity than the surrounding forest, resulting in consequences to both their physiology and encounter with novel parasites (Jessop *et al.,* 2012; Smyth *et al.,* 2014; Wells *et al.,* 2018). Previous research indicates that the relative abundance of rodents in the Kinabatangan floodplain is not different between forest and the surrounding OPPs (Guerrero-Sanchez *et al.,* 2022). Nonetheless, in this study, rodents represented the dominant prey group collected from the vomit of plantation lizards, with only a few records from the forest. A study on habitat use of Bornean leopard cats (*Prionailurus bengalensis*) suggests that the homogeneous structure of oil palm habitats facilitates the capture of rodents, over that in natural forests where habitat heterogeneity provides shelter and protection for small mammals (Rajaratnam *et al.,* 2007). Despite the similar abundances found in both habitats, the higher number of rodents recorded from the stomach content in plantation lizards, compared to those in the forest, is consistent with the aforementioned study.

### BC and biochemistry

Our main assumption was based on the hypothesis that monitor lizards inhabiting OPP would have a higher BC index, as well as higher levels of diet-related biomarkers. However, our results showed the opposite regarding both total and LDL-Ch c, which were significantly higher in forested areas. This could be influenced by the low levels of LDL-Ch we found in the Kuril estate, which also presents the highest species richness in the dietary inventory among oil palm sites.

The interaction between two different habitats (natural forest and OPP, in this case) can affect, either positively or negatively, the composition and structure of animal communities, as well as species distribution and behaviour (Potts *et al.*, 2016). For Asian water monitor lizards, variations in prey community structure between edge and interior areas also influence individual movement and population distribution (Jessop *et al.,* 2012). A previous study showed no differences in lizards’ abundance and body size between OPP and forested areas (Guerrero-Sanchez *et al.,* 2021). However, the distribution of abundance and body size in narrow patches of forest showed a tendency towards the mean values observed in plantation sites, suggesting an influence of anthropogenic habitats on adjacent forest, i.e. an edge effect. Here, this edge effect extends to the physiology of the population, where both BC and lipid levels of lizards in Lot 5 are similar to lizards inhabiting the adjacent oil palm estate (Hillco). However, the dietary inventory does not present similarities between these two sites. Hence, the edge effect in this case would not necessarily apply to the distribution of both prey and lizards, but it could be related to the physiology of the prey. An experimental study carried out by Mayntz and Toft (2001), for example, concluded that variations in wolf spiders’ metabolites depended on the nutrient composition of the prey’s diet. The effect of prey’s food on the predator’s nutrient intake is worth considering in future studies.

From an animal’s perspective, physiological biomarkers are a valuable tool to understand environmental changes (Cooke *et al.,* 2013). Not only are they highly sensitive to environmental alterations, but also their variations are often related to fitness components that drive population persistence (Bergman *et al.,* 2019). Lipids, triglycerides and cholesterol are important metabolites derived from the metabolism of carbohydrates and lipids (Meyer and Harvey, 2004). As a secondary source of energy, their levels are associated not only with food quality but also with individuals’ activity patterns, where sedentary behaviour can lead to an energy demand–intake imbalance. To explore the effects of such behaviour on BC and biochemical levels, we incorporated previous information of home range sizes for the same population (Guerrero-Sanchez *et al.,* 2022), showing that lizards with larger home ranges spend more time roaming between different core areas than those inhabiting OPPs. Our findings suggest that sedentary behaviour, coupled with low diverse diets, specially based on small mammals (i.e. rodents; Kaneko and Maruyama, 2005), leads to higher levels of LDL-Ch, known to be associated with cardiovascular disorders (Meyer and Harvey, 2004). On the other hand, high values of HDL-Ch, cholesterol, and triglycerides, are associated with a higher dietary diversity in lizards with larger home ranges, and are more likely to be associated with the demand of energy required to constantly roam between core areas. Similarly to the LDL-Ch, these two lipids are associated with energy metabolism but can be metabolized faster when energy demand exceeds the intake.

The effects of habitat type on electrolytes, such as potassium and chloride, could be a consequence of the ingestion of processed food, either directly or through the food web (Jessop *et al.,* 2012). Total proteins, on the other hand, seem to increase with dietary diversity, but the effect is more intense in oil palm habitats. As a carnivorous generalist, the diet of a monitor lizard has a high protein content. However, our findings suggest a stronger correlation in OPP than in forested areas, which could be explained by the biomass intake rather than by the actual dietary diversity. In addition, higher levels of globulin in lizards inhabiting OPP, compared with those living in forest sites, could be an effect likely determined by the higher exposure to pathogens (i.e. parasitic helminths) in oil palm plantations than in forest sites. Overall, our findings regarding home range size highlight the importance of improving both size and quality of forest patches within OPP, such as high conservation value areas, in order to increase prey diversity and promote larger animal mobility (Guerrero-Sanchez *et al.,* 2022). Not only would these changes in land use management benefit the physiological health of the Asian water monitor lizard population, and other generalist species in the area, but it also has been suggested to benefit the survival of other species such as orangutans (*Pongo* spp.; Ancrenaz *et al.,* 2021).

### Diet and parasite community

Diet is the host trait most strongly associated with the composition of helminth communities (Leung and Koprivnikar, 2019). Soil-transmitted parasites, such as capillarids, trichurids, strongyles, *Strongyloides* spp. and oxyurids, have simple and direct life cycles that can be transmitted either from the environment or through predation, while other parasites such as spirurids, cestodes and trematodes have complex life cycles and need one or more intermediate hosts to develop into infecting stages (Galaktionov and Dobrovolskij, 2003). In this study, diets that were more diverse were associated with higher prevalence of trichurids and strongyles, which forest lizards might encounter while foraging in the forest. Less diverse diets, on the other hand, were related to higher prevalence of *Strongyloides* spp., oxyurids and capillarids, which are parasites prevalent in rodents (Wells *et al.,* 2007; Frias, personal observation) that could have been transmitted trophically to the lizards.

The higher presence of invertebrates in the stomach of lizards in forested areas, compared with those in OPP, is consistent with the presence of trematodes and cestodes in natural habitats. However, a highly diverse dietary content also influences the low prevalence of such parasites. Although lizards generally inhabit partially inundated habitats, the presence of two large oxbow lakes in Lot 6 can favour the life cycle of digenean trematodes, which have free-living aquatic stages, and requires specific conditions for successful transmission (Galaktionov and Dobrovolskij, 2003). At the same time, lizards living in aquatic habitats may be more likely to feed on animals that contain trematode infective stages (Combes *et al.,* 1994). We should not discard the presence of a research station in Lot 6, its intense human activity in the area, and its role in the high parasite prevalence, very similar to those found in oil palm estates.

Anthropogenic habitats can alter host–parasite interactions and lead to either increased or decreased infection risk (Patz *et al.,* 2000; Gillespie and Chapman, 2006; Lafferty *et al.,* 2006; Cardoso *et al.,* 2016; Bonnell *et al.,* 2018). Individuals having diverse parasite communities maintain a healthy balance among parasite populations (Lafferty *et al.,* 2006). But when such balance is disturbed, it leads to the increase of certain parasite groups and negatively impacts the individual fitness and the population survival (Frias and MacIntosh, 2019). By decreasing their foraging activities in the forest, plantation lizards also decrease their encounters with parasites with complex life cycles. Similarly, having rodents as a predictable food source can increase the prevalence of helminths transmitted through prey ingestion in plantation lizards (Lafferty *et al.,* 2006; Dunne *et al.,* 2013; Leung and Koprivnikar, 2019). We also observed a significant association between BC and both parasite richness and intensity of infection in plantation lizards, where smaller lizards hosted fewer parasite species and shed more parasite infective stages into the environment. This observation suggests that homogeneous diets may alter host parasite communities and potentially impact individual fitness (Lafferty *et al.,* 2006; Frias and MacIntosh, 2019).

### The *fast-food effect*: the role of dietary diversity and sedentarism on population health

Fast-food, as we know it, is usually associated with hypercaloric meals that are quick, convenient and low priced. Long-term consumption of fast-food is also associated with obesity and cardiovascular disorders (Rosenheck, 2008; Alter and Eny, 2005), and the proximity of fast-food restaurants to schools has been linked to obesity in teenagers in the United States (Davis and Carpenter, 2009). Similarly, human-dominated landscapes, especially industrial crops and farms, offer neighbouring wildlife abundant food resources that are easily accessible, convenient, and have similar health consequences to animal populations (Murray *et al.,* 2015), hence a *fast-food effect*. Although the impact of the *fast-food effect* may not be immediately noticeable for lizards and other reptiles, it could pose a risk for mammals and birds that have adapted to similar landscapes and are more sensitive to physiological changes (Becker *et al.,* 2015; Murray *et al.,* 2015; Murray *et al.,* 2016). The Asian water monitor lizard is a widespread, highly adaptable and long-lived species commonly found in human-altered landscapes. These life history traits make it a fitting model species to help us understand the physiological threats posed by changing ecosystems.

Fast-food consumption in humans is a complex health issue that not only involves obesity and cardiovascular disorders but also implies a cascade of social, economic and cultural causes and consequences (Rosenheck, 2008; Davis and Carpenter, 2009). Likewise, the *fast-food effect* in wildlife involves a complex series of elements that need to be understood by observing different aspects of wildlife ecology (i.e. prey abundance and distribution, human subsidies, demography, distribution, activity patterns of target species, etc.) and not only by comparing populations in different habitats. Aside from dietary diversity and movement ranges, other important elements to consider are biomass intake, including the nutritional properties of prey, and parasites present in prey communities. While the first element would offer a more complete picture of how energy intake is used and metabolized by predators, a wider knowledge of parasite communities infecting prey would help us understand transmission pathways through food webs (Lafferty *et al.,* 2006). Additional demographic and longitudinal information would be important complements to evaluate the impact of the *fast-food effect* on individual fitness.

The metabolic responses of lizards and the changes in parasite community composition, influenced by a diet derived from landscape alterations, suggest a decreased physiological status and fitness in the wildlife community in the area. The abundance and high catchability rate of Asian water monitor lizards (Guerrero-Sanchez *et al.,* 2021) allowed us to generate more data than it would have been possible by targeting other wildlife species living in similar habitats, which are more cryptic or have a lower catchability rate, such as leopard cats (*P. bengalensis*), civets (*Viverra tangalunga* and *Paradoxurus hermaphroditus*), macaques (*Macaca fascicularis* and *Macaca. nemestrina*), Bornean sun bears (*Helarctos malayanus*) and bearded pigs (*Sus barbatus*), among others. Such information provides a hint towards the hidden consequences that living in disturbed ecosystems can have for other species as well.

Finally, this study presents the most robust data on blood biochemistry for the Asian water monitor lizard in the wild. Although none of the sampled individuals showed signs of being unhealthy, it remains to be explored whether these metabolite levels lay or not within healthy ranges for populations living in the forest and in plantations. To our knowledge, there is no reference database that allows for an accurate comparison of our results. The International Species Information System (https://www.species360.org/) is the most comprehensive biometric database for animal information; however, it is mostly composed of samples from captive animals with different dietary schemes. Hence, we recommend caution when using the information presented here as reference for the species.

## Supplementary Material

Web_Material_coad055
