# Supplementary material for: The fast-food effect: costs of being a generalist in a human-dominated landscape
Source: Conserv Physiol. 2023 Aug 14;11(1):coad055. doi: 10.1093/conphys/coad055 (PMC10427121; doi:10.1093/conphys/coad055)

Supplementary material

Supplementary Table 1. Mean value of body measurements and biochemical parameters analyzed for Asian water monitor lizards. Values presented as mean ±SD; (min - max), and sampling size (n).

Parameter	Lot 5	Lot 6	Lot 7	Forest	Hillco	Kopi	Kuril	Plantation
**Body measurements**								
Weight (Kg.)	5.98±3.77 (1.5-16.5) n=24	5.76±4.98 (1.5-22) n=40	5.27±4.49 (1-18) n=28	5.67±4.5 (1-22) n=92	6.15±3.33 (1.5-11.6) n=28	6.05±3.63 (2-15) n=53	6.02±3.65 (2-17) n=25	6.07±3.52 (1.5-17) n=106
Body condition	0.39±0.14 (0.11-0.65) n=24	0.34±0.17 (0.03-0.67) n=40	0.35±0.13 (0.17-0.63) n=28	0.35±0.15 (0.03-0.67) n=92	0.39±0.12 (0.11-0.57) n=28	0.39±0.13 (0.17-0.60) n=53	0.39±0.12 (0.18-0.63) n=25	0.39±0.12 (0.11-0.63) n=106
**Lipids**								
Cholesterol (mmol/L)	2.21±0.67 (1.20-3.60) n=24	2.04±0.63 (1.20-4.70) n=40	1.89±0.70 (0.80-3) n=28	2.04±0.67 (0.80-4.70) n=92	2.17±0.84 (0.80-4.20) n=28	1.43±0.82 (0.07-5.0) n=52	2.17±0.54 (1.30-3.70) n=24	1.8±0.85 (0.07-5.0) n=104
LDL-Ch (mmol/L)	1.68±0.85 (0.1-3.25) n=21	1.59±0.62 (0.22-2.62) n=31	1.79±2.14 (0.66-11.6) n=25	1.68±1.34 (0.1-11.6) n=77	1.76±0.82 (0.29-3.44) n=24	0.97±0.66 (0.1-2.72) n=42	1.73±0.71 (0.25-3.53) n=19	1.36±0.81 (0.1-3.53) n=85
HDL-Ch (mmol/L)	0.18±0.14 (0.1-0.59) n=22	0.11±0.03 (0.1-0.22) n=32	0.19±0.20 (0.1-0.82) n=27	0.15±0.14 (0.1-0.82) n=81	0.13±0.07 (0.1-0.4) n=25	0.14±0.06 (0.1-0.4) n=47	0.11±0.04 (0.1-0.29) n=24	0.13±0.06 (0.1-0.4) n=96
Triglycerides (mmol/L)	1.62±2.58 (0.05-9.92) n=23	2.18±3.57 (0.1-15.26) n=34	2.47±4.54 (0.05-20.87) n=28	2.12±3.67 (0.05-20.87) n=85	1.33±2.03 (0.05-9.13) n=25	0.98±1.73 (0.05-8.99) n=49	1.97±2.59 (0.06-10.28) n=24	1.31±2.06 (0.05-10.28) n=98

Supplementary Table 1 (cont.). Mean value of body measurements and biochemical parameters analyzed for Asian water monitor lizards. Values presented as mean ±SD; (min - max), and sampling size (n).

Parameter	Lot 5	Lot 6	Lot 7	Forest	Hillco	Kopi	Kuril	Plantation
**Proteins**								
Total protein (g/L)	78.1±8.33 (62-88) n=13	76.6±9.44 (56-96) n=39	75.1±7.23 (63-83) n=15	76.6±8.71 (56-96) n=67	77.6±10.8 (56-106) n=20	83.6±6 (69-94) n=21	79.5±6.49 (65-90) n=24	80.3±8.18 (56-106) n=65
Albumin (g/L)	26.1±2.9 (20-30) n=13	25.5±4.03 (10-34) n=39	25.3±2.25 (21-28) n=15	25.6±3.47 (10-34) n=67	24.4±5.4 (10-30) n=20	27.6±1.86 (23-31) n=21	26.8±2.56 (22-34) n=24	26.3±3.72 (10-34) n=65
Globulin (g/L)	52±5.76 (42-60) n=13	51.3±7.28 (36-66) n=39	49.9±5.76 (39-57) n=15	51.1±6.64 (36-66) n=67	53.8±13.2 (36-100) n=20	56±4.32 (46-65) n=21	52.8±4.94 (43-60) n=24	54.1±8.24 (36-100) n=65
Uric acid (mmol/L)	0.67±0.31 (0.27-1.44) n=24	0.65±0.29 (0.17-1.49) n=40	0.66±0.45 (0.1-2.42) n=28	0.66±0.35 (0.1-2.42) n=92	0.60±0.22 (0.22-1.07) n=28	0.5±0.31 (0.13-1.33) n=52	0.65±0.30 (0.17-1.53) n=24	0.56±0.29 (0.13-1.53) n=104
**Electrolytes**								
Sodium (mmol/L)	157±4.84 (146-164) n=13	160±5.49 (152-172) n=39	157±4.33 (152-167) n=15	159±5.28 (146-172) n=67	160±5.65 (145-167) n=20	161±4.31 (152-170) n=21	161±7.07 (136-171) n=25	161±5.84 (136-171) n=66
Potassium (mmol/L)	31.8±8.16 (24.4-56.1) n=13	30.6±5.48 (20.4-43) n=34	31.8±6.56 (25.7-50.4) n=12	31.1±6.28 (20.4-56.1) n=59	33.3±7.9 (24.7-58) n=19	30.7±3.7 (24.4-37.2) n=21	29.9±7.49 (22.7-50.7) n=25	31.2±6.71 (22.7-58) n=65
Chloride (mmol/L)	99.3±6.56 (80-109) N=13	102±6.03 (85-112) n=39	103±4.51 (96-111) n=15	102±5.9 (80-112) n=67	105±6.21 (91-116) n=20	103±5.48 (87-110) n=21	103±5.75 (89-111) n=25	104±5.83 (87-116) n=66

Supplementary Table 2. Differences in body condition and biochemical markers among habitat type, sites, and sites grouped per habitat type.

Marker	Variable	Xi^2	p
**Body condition**	Habitat	**0.06**	**0.06**
	Sites	0.11	0.31
	Forest sites	0.05	0.35
	Oil palm plantation sites	0.00	0.96
**Total cholesterol**	Habitat	**2.80**	**0.03**
	Sites	**18.36**	**< 0.001**
	Forest sites	1.35	0.21
	Oil palm plantation sites	**14.20**	**< 0.001**
**LDL - cholesterol**	Habitat	**1.77**	**0.06**
	Sites	**9.16**	**< 0.001**
	Forest sites	0.20	0.84
	Oil palm plantation sites	**7.18**	**< 0.001**
**HDL - cholesterol**	Habitat	0.14	0.38
	Sites	**2.61**	**0.01**
	Forest sites	**2.01**	**0.02**
	Oil palm plantation sites	0.47	0.13
**Triglycerides**	Habitat	3.33	0.25
	Sites	16.49	0.26
	Forest sites	1.95	0.71
	Oil palm plantation sites	11.21	0.08
**Total proteins**	Habitat	**446.66**	**0.01**
	Sites	**898.17**	**0.03**
	Forest sites	60.67	0.68
	Oil palm plantation sites	**390.84**	**0.04**
**Albumin**	Habitat	18.09	0.24
	**Sites**	**136.53**	**0.05**
	Forest sites	**4.85**	**0.82**
	Oil palm plantation sites	**113.59**	**0.01**
**Globulin**	Habitat	**0.10**	**0.02**
	Sites	0.17	0.10
	Forest sites	0.01	0.70
	Oil palm plantation sites	0.05	0.25

Supplementary Table 2 (cont.) Differences in body condition and biochemical markers among habitat type, sites, and sites grouped per habitat type.

Marker	Variable	Xi^2	p
**Uric acid**	**Habitat**	**1.15**	**0.04**
	Sites	2.57	0.11
	Forest sites	0.03	0.96
	Oil palm plantation sites	1.39	0.08
**Sodium**	Habitat	81.14	0.11
	Sites	274.49	0.11
	Forest sites	153.03	0.06
	Oil palm plantation sites	40.32	0.56
**Potassium**	Habitat	0.00	0.96
	Sites	0.15	0.50
	Forest sites	0.02	0.77
	Oil palm plantation sites	0.14	0.15
**Chloride**	Habitat	104.14	0.08
	Sites	303.33	0.11
	Forest sites	113.72	0.19
	Oil palm plantation sites	85.47	0.28

Supplementary Table 3. Generalized estimation equations (GEE) models outcomes for the effect of body condition and dietary diversity, as estimated with the Shannon-Wiener Index (H'), on the biochemical markers of Asian water monitor lizards based on habitat type.

**Body condition**	**ß**	**Std. Err.**	**W**	**p**
(Intercept)	2.531	0.2690	88.45	<2e-16
Dietary diversity : forest	0.1408	0.1507	0.87	0.35
Dietary diversity : OPP	0.0364	0.2558	0.02	0.89
**Total cholesterol**	**ß**	**Std. Err.**	**W**	**p**
(Intercept)	2.545	0.301	71.28	<2e-16
Dietary diversity : forest	-0.224	0.155	2.10	0.15
Dietary diversity : OPP	-0.555	0.347	2.56	0.11
**LDL – cholesterol**	**ß**	**Std. Err.**	**W**	**p**
(Intercept)	2.226	0.332	44.86	**2.1e-11**
Dietary diversity : forest	-0.336	0.162	4.30	**0.038**
Dietary diversity : OPP	-0.801	0.332	5.84	**0.016**
**HDL - cholesterol**	**ß**	**Std. Err.**	**W**	**p**
(Intercept)	0.3884	0.0967	16.13	**5.9e-05**
Dietary diversity : forest	-0.1187	0.0518	5.25	**0.022**
Dietary diversity : OPP	-0.2020	0.0776	6.78	**0.009**
**Triglycerides**	**ß**	**Std. Err.**	**W**	**p**
(Intercept)	2.4601	0.4725	27.10	1.9e-07
Dietary diversity : forest	0.0671	0.2789	0.06	0.809
Dietary diversity : OPP	-1.4583	0.4665	9.77	**0.0018**
**Total protein**	**ß**	**Std. Err.**	**W**	**p**
(Intercept)	63.23	4.42	204.41	< 2e-16
Dietary diversity : forest	5.33	2.32	5.29	**0.02144**
Dietary diversity : OPP	21.55	5.82	13.73	**0.00021**
**Albumin**	**ß**	**Std. Err.**	**W**	**p**
(Intercept)	22.56	2.53	79.56	<2e-16
Dietary diversity : forest	1.43	1.20	1.41	0.234
Dietary diversity : OPP	3.64	2.05	3.14	0.077
**Globulin**	**ß**	**Std. Err.**	**W**	**p**
(Intercept)	52.809	3.164	278.62	<2e-16
Dietary diversity : forest	-0.729	1.593	0.21	0.65
Dietary diversity : OPP	1.231	2.748	0.20	0.65

Supplementary Table 3 (cont.). Generalized estimation equations (GEE) models outcomes for the effect of body condition and dietary diversity, as estimated with the Shannon-Wiener Index (H'), on the biochemical markers of Asian water monitor lizards based on habitat type.

**Uric acid**	**ß**	**Std. Err.**	**W**	**p**
(Intercept)	0.6352	0.1071	35.19	3e-09
Dietary diversity : forest	0.0163	0.0537	0.09	0.76
Dietary diversity : OPP	-0.0429	0.1201	0.13	0.72
**Sodium**	**ß**	**Std. Err.**	**W**	**p**
(Intercept)	163.13	2.37	4722.59	<2e-16
Dietary diversity : forest	-1.23	1.14	1.17	0.28
Dietary diversity : OPP	-2.79	1.98	1.99	0.16
**Potassium**	**ß**	**Std. Err.**	**W**	**p**
(Intercept)	35.85	2.50	205.03	<2e-16
Dietary diversity : forest	-2.23	1.19	3.50	0.0613
Dietary diversity : OPP	-5.25	1.98	6.99	**0.0082**
**Chloride**	**ß**	**Std. Err.**	**W**	**P**
(Intercept)	102.58	4.09	630.23	<2e-16
Dietary diversity : forest	-2.83	2.02	1.96	0.16
Dietary diversity : OPP	-5.19	3.95	1.73	0.19

Supplementary Table 4. Generalized estimation equations (GEE) models outcomes for the effect of body condition and dietary diversity, as estimated with the Shannon-Wiener Index (H'), on the biochemical markers of Asian water monitor lizards based on home range size. Home range (HR) was estimated using Local Convex Hull and expressed in Km^2.

**Body condition**	**ß**	**Std. Err.**	**W**	**p**
(Intercept)	0.4917	0.0423	135.29	< 2e-16
Dietary diversity (H')	0.0596	0.0294	4.11	**0.0427**
Home range	0.2648	0.0677	15.29	**9.2e-05**
Dietary diversity * HR	-0.1221	0.0454	7.22	**0.0072**
**Total cholesterol**	**ß**	**Std. Err.**	**W**	**P**
(Intercept)	3.839	1.609	5.69	0.017
Dietary Diversity (H')	-1.411	0.797	3.14	0.077
Home range	-3.091	2.999	1.06	0.303
Dietary diversity * HR	1.712	1.408	1.48	0.224
**LDL - cholesterol**	**ß**	**Std. Err.**	**W**	**p**
(Intercept)	-1.158	0.427	7.37	0.0066
Dietary Diversity (H')	1.566	0.318	24.27	**8.4e-07**
Home range	0.843	0.434	3.77	0.0521
Dietary diversity * HR	-0.992	0.280	12.54	**0.0004**
**HDL - cholesterol**	**ß**	**Std. Err.**	**W**	**p**
(Intercept)	-0.493	0.642	0.59	0.4421
Dietary Diversity (H')	-0.782	0.260	9.07	**0.0026**
Home range	-3.234	1.479	4.78	**0.0287**
Dietary diversity * HR	1.425	0.649	5.00	**0.0254**
**Triglycerides**	**ß**	**Std. Err.**	**W**	**p**
(Intercept)	3.868	1.946	3.95	0.0469
Dietary Diversity (H')	-2.949	0.946	9.71	**0.0018**
Home range	-14.335	3.518	16.61	**4.6e-05**
Dietary diversity * HR	7.087	1.685	17.68	**2.6e-05**
**Uric acid**	**ß**	**Std. Err.**	**W**	**p**
(Intercept)	3.67	2.84	1.67	0.20
Dietary Diversity (H')	-0.61	1.62	0.14	0.71
Home range	-9.53	16.16	0.35	0.56
Dietary diversity * HR	-4.59	7.45	0.38	0.54
**Total protein**	**ß**	**Std. Err.**	**W**	**p**
(Intercept)	89.39	9.09	96.62	<2e-16
Dietary Diversity (H')	-9.42	6.62	2.02	0.15
Home range	3.92	16.42	0.06	0.81
Dietary diversity * HR	1.59	10.89	0.02	0.88

Supplementary Table 4 (cont.). Generalized estimation equations (GEE) models outcomes for the effect of body condition and dietary diversity, as estimated with the Shannon-Wiener Index (H'), on the biochemical markers of Asian water monitor lizards based on home range size. Home range was estimated using Local Convex Hull and expressed in Km^2.

**Albumin**	**ß**	**Std. Err.**	**W**	**p**
(Intercept)	53.80	14.76	13.28	0.00027
Dietary Diversity (H')	-19.54	9.66	4.09	**0.04303**
Home range	-18.90	28.17	0.45	0.50216
Dietary diversity * HR	14.10	14.10	1.00	0.31744
**Globulin**	**ß**	**Std. Err.**	**W**	**p**
(Intercept)	3.4887	0.1641	451.89	<2e-16
Dietary Diversity (H')	0.3109	0.0872	12.72	**<0.001**
Home range	0.5508	0.4557	1.46	0.22678
Dietary diversity * HR	-0.3451	0.2102	2.69	0.10071
**Sodium**	**ß**	**Std. Err.**	**W**	**p**
(Intercept)	136.42	20.07	46.19	1.1e-11
Dietary Diversity (H')	6.27	9.21	0.46	0.50
Home range	3.11	36.15	0.01	0.93
Dietary diversity * HR	2.15	16.69	0.02	0.90
**Potassium**	**ß**	**Std. Err.**	**W**	**p**
(Intercept)	3.5130	0.5684	38.20	6.4e-10
Dietary Diversity (H')	0.0535	0.2610	0.04	0.835
Home range	-0.6470	1.0342	0.39	0.530
Dietary diversity * HR	0.1916	0.4769	0.16	0.690
**Chloride**	**ß**	**Std. Err.**	**W**	**P**
(Intercept)	101.31	7.32	191.49	<2e-16
Dietary Diversity (H')	2.32	5.08	0.21	0.65
Home range	-25.25	16.34	2.39	0.12
Dietary diversity * HR	11.58	9.17	1.60	0.21

Supplementary Table 5. Outputs of generalized estimation equation (GEE) on the effect of body condition index and dietary diversity, as estimated with the Shannon-Wiener Index (H'), on the prevalence of each parasite taxonomic group.

**Parasite richness (both habitats)**				
**Variable**	**ß**	**Std. Err.**	**W**	**p**
(Intercept)	0.0232	0. 5585	0.00	0.97
Body condition	0.8389	0.9104	0.85	0.36
Diet diversity (H')	0.0568	0.2395	0.06	0.81
**Parasite richness (forest)**				
**Variable**	**ß**	**Std. Err.**	**W**	**p**
(Intercept)	-3.415	2.703	1.60	0.21
Body condition	-0.451	1.521	0.09	0.77
Diet diversity (H')	1.922	1.224	2.39	0.12
**Parasite richness (oil palm plantation)**				
**Variable**	**ß**	**Std. Err.**	**W**	**p**
(Intercept)	-0.150	0.512	0.09	0.77
Body condition	2.231	0.739	9.12	0.002
Diet diversity (H')	-0.357	0.272	1.72	0.19
**Prevalence (overall)**				
**Variable**	**ß**	**Std. Err.**	**W**	**p**
(Intercept)	0.998	0.247	16.27	5.5e-05
Body condition	0.273	0.200	1.86	0.17
Diet diversity (H')	-0.152	0.119	1.65	0.20
***Capillaria* spp. (prevalence)**				
**Variable**	**ß**	**Std. Err.**	**W**	**p**
(Intercept)	2.497	0.362	47.45	5.6e-12
Body condition	-0.125	0.113	1.22	0.27
Diet diversity (H')	-0.289	0.196	2.17	0.14
**Oxyurida spp. (prevalence)**				
**Variable**	**ß**	**Std. Err.**	**W**	**p**
(Intercept)	1.842	0.105	306.08	<2e-16
Body condition	0.163	0.172	0.90	0.34
Diet diversity (H')	-0.102	0.126	0.66	0.42
***Physaloptera* spp. (prevalence)**				
**Variable**	**ß**	**Std. Err.**	**W**	**p**
(Intercept)	0.661	0.3284	4.05	0.0442
Body condition	0.017	0.0611	0.08	0.7774
Diet diversity (H')	-0.665	0.2529	6.91	**0.0086**

Supplementary Table 5 (cont.). Outputs of generalized estimation equation (GEE) on the effect of body condition index and dietary diversity, as estimated with the Shannon-Wiener Index (H'), on the prevalence of each parasite taxonomic group.

***Spirurida* spp. (prevalence)**				
**Variable**	**ß**	**Std. Err.**	**W**	**p**
(Intercept)	-1.029	0.702	2.15	0.14
Body condition	0.159	-0.134	1.42	0.23
Dietary diversity (H')	0.394	0.335	1.39	0.24
**Strongylida spp. (prevalence)**				
**Variable**	**ß**	**Std. Err.**	**W**	**p**
(Intercept)	1.886	0.435	18.80	1.4e-05
Body condition	-0.155	0.241	0.41	0.52
Dietary diversity (H')	-0.307	0.213	2.07	0.15
***Strongyloides* spp. (prevalence)**				
**Variable**	**ß**	**Std. Err.**	**W**	**p**
(Intercept)	1.709	0.2624	42.39	7.5e-11
Body condition	-0.049	-0.0427	1.32	0.251
Dietary diversity (H')	-0.374	0.1978	3.59	**0.058**
***Trichuris* spp. (prevalence)**				
**Variable**	**ß**	**Std. Err.**	**W**	**p**
(Intercept)	-0.713	0.5582	1.63	0.202
Body condition	0.021	0.1092	0.04	0.850
Dietary diversity (H')	0.594	0.2758	4.64	**0.031**

Supplementary material

Section 2

Fit of distribution for the biochemical values, body condition and parasite data, by maximum likelihood estimation

Body condition

Summary:

**min: 0.03 max: 0.67**	**median: 0.36**
**mean: 0.373**	**sample sd: 0.1.36**
**sample skewness: 0.105**	**sample kurtosis: 2.24**

Candidates: (Best fit distribution in marked in bold)

**Normal**	**Mean (SE)**	**Sd (SE)**	**Loglikelihood**	**AIC**	**BIC**
	**0.373 (0.01)**	**0.14 (0.01)**	**114**	**-223**	**-217**

Gamma	**Shape (SE)**	**Rate (SE)**	**Loglikelihood**	**AIC**	**BIC**
	6.34 (0.62)	17.00 (1.73)	108	-212	-206

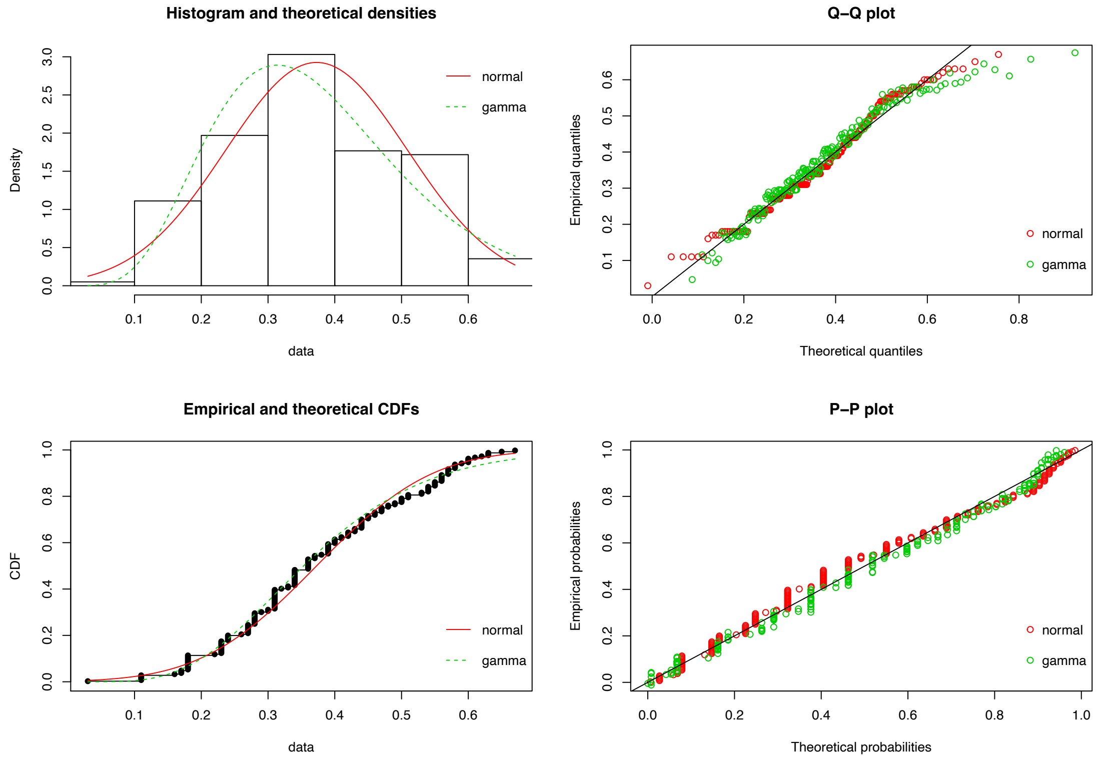

Cholesterol

Summary:

**min: 0.07 max: 5**	**median: 1.8**
**mean: 1.914**	**sample sd: 0.7739**
**sample skewness: 0.7021**	**sample kurtosis: 4.363**

Candidates: (Best fit distribution in marked in bold)

**Normal**	**Mean (SE)**	**Sd (SE)**	**Loglikelihood**	**AIC**	**BIC**
	**1.91 (0.06)**	**0.77 (0.04)**	**-227.9**	**459.8**	**466.3**

Gamma	**Shape (SE)**	**Rate (SE)**	**Loglikelihood**	**AIC**	**BIC**
	5.36 (0.55)	2.8 (0.29)	-228.1	460.2	466.7

Log normal	**LogMean**	**LogSd**	**Loglikelihood**	**AIC**	**BIC**
	0.55 (0.03)	0.49 (0.02)	-254.4	494.8	501.3

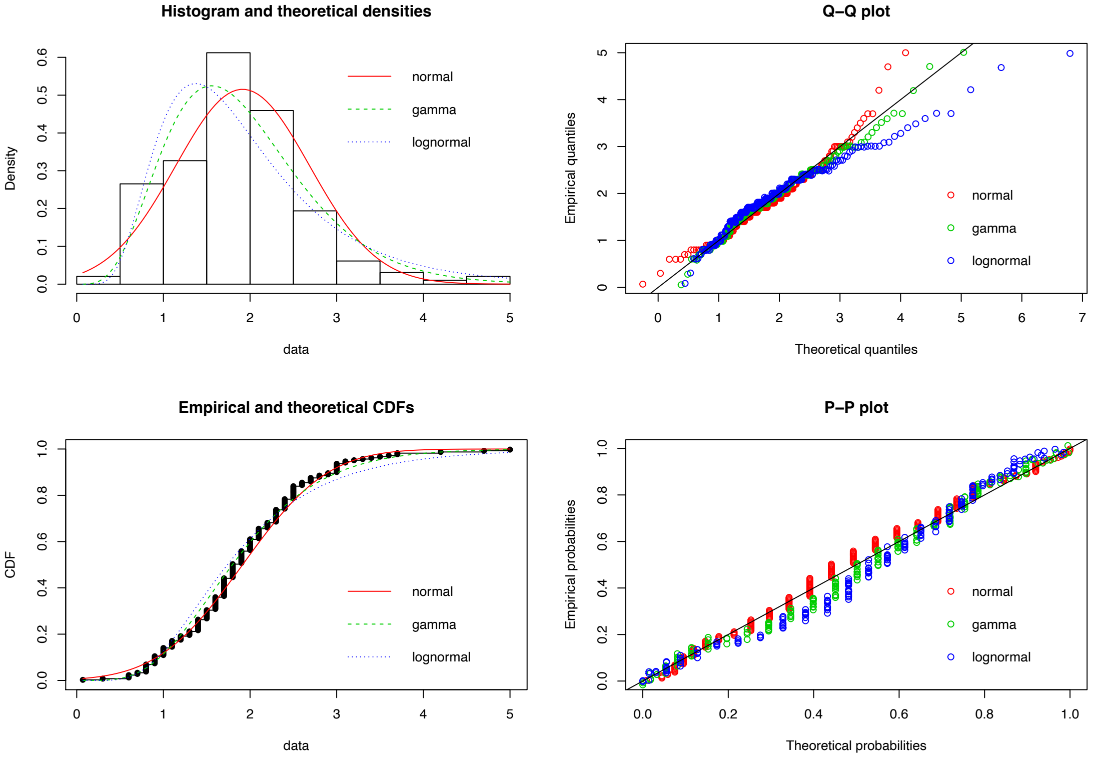

Low Density Lipoprotein cholesterol

Summary:

**min: 0.1 max: 11.6**	**median: 1.515**
**mean: 1.514**	**sample sd: 1.099**
**sample skewness: 4.805**	**sample kurtosis: 44.39**

Candidates: (Best fit distribution in marked in bold)

Normal	**Mean (SE)**	**Sd (SE)**	**Loglikelihood**	**AIC**	**BIC**
	1.51 (0.09)	1.1 (0.06)	-245.2	494.3	500.5

**Gamma**	**Shape (SE)**	**Rate (SE)**	**Loglikelihood**	**AIC**	**BIC**
	**2.38 (0.25)**	**1.57 (0.18)**	**-201.6**	**407.3**	**413.4**

Log normal	**LogMean**	**LogSd**	**Loglikelihood**	**AIC**	**BIC**
	0.19 (0.06)	0.74 (0.04)	-212.5	429.1	435.3

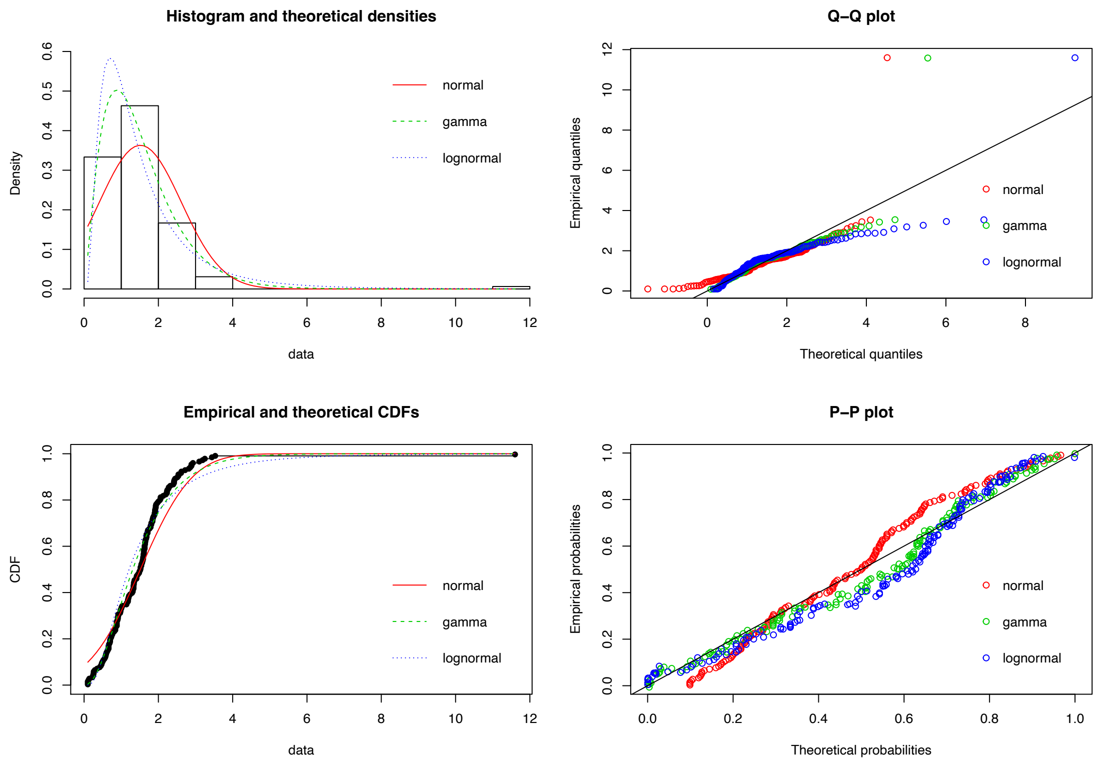

High Density Lipoprotein cholesterol

Summary:

**min: 0.1 max: 0.82**	**median: 0.1**
**mean: 0.1408**	**sample sd: 0.106**
**sample skewness: 4.099**	**sample kurtosis: 22.28**

Candidates: (Best fit distribution in marked in bold)

Gamma	**Shape (SE)**	**Rate (SE)**	**Loglikelihood**	**AIC**	**BIC**
	4.04 (0.41)	28.73 (3.13)	235	-466.1	-459.7

**Log normal**	**LogMean**	**LogSd**	**Loglikelihood**	**AIC**	**BIC**
	**-2.01 (0.03)**	**0.42 (0.02)**	**269.9**	**-535.8**	**-529.4**

Beta	**Shape 1**	**Shape 2**	**Loglikelihood**	**AIC**	**BIC**
	2.56 (0.26)	14.74 (1.60)	205.2	-406.4	-400

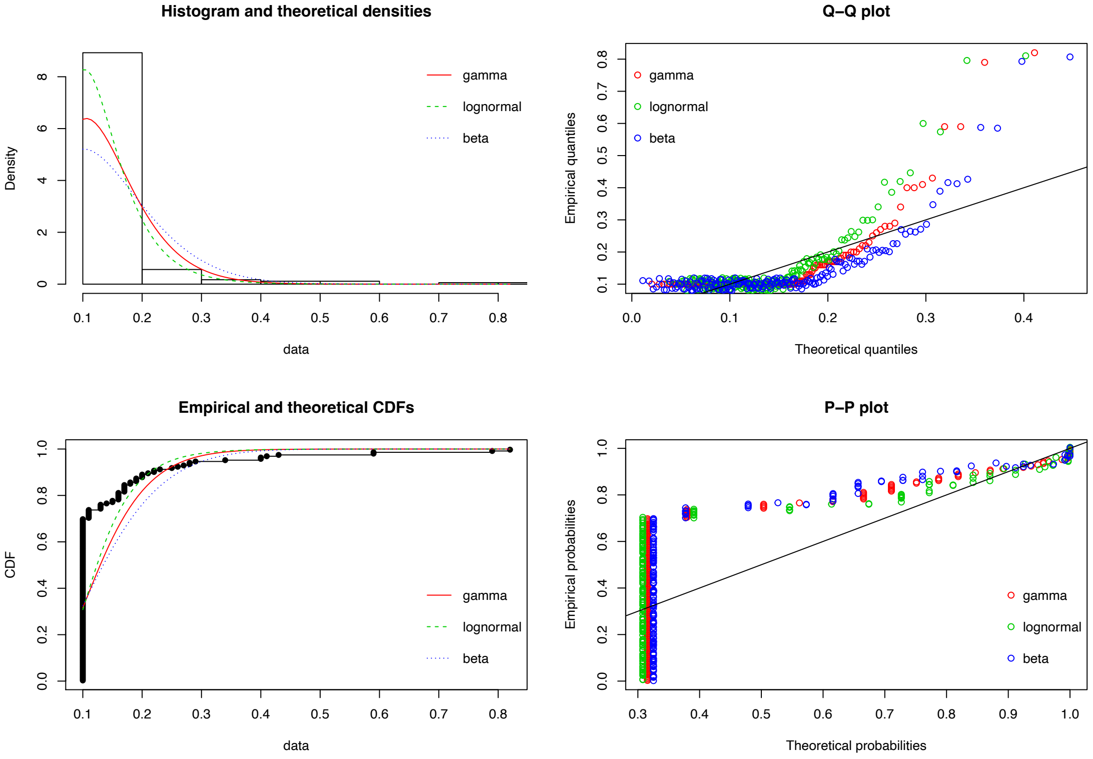

Triglycerides

Summary:

**min: 0.05 max: 20.87**	**median: 0.44**
**mean: 1.688**	**sample sd: 2.935**
**sample skewness: 3.134**	**sample kurtosis: 15.45**

Candidates: (Best fit distribution in marked in bold)

Gamma	Shape (SE)	Rate (SE)	Loglikelihood	AIC	BIC
	0.520 (0.05)	0.31 (0.04)	-244.4	492.8	499.2

**Log normal**	**LogMean**	**LogSd**	**Loglikelihood**	**AIC**	**BIC**
	**-0.69 (0.12)**	**1.6 (0.08)**	**-219.6**	**443.2**	**449.7**

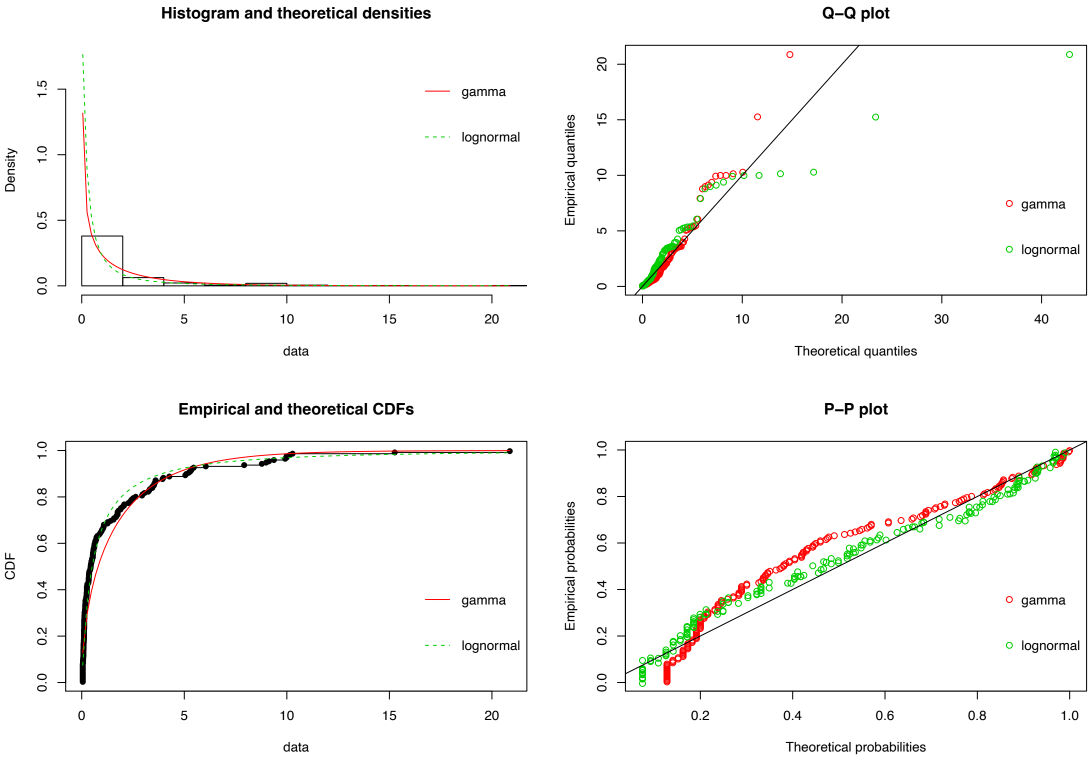

Total proteins

Summary:

**min: 56 max: 106**	**median: 78.5**
**mean: 78.39**	**sample sd: 8.589**
**sample skewness: 0.02671**	**sample kurtosis: 3.306**

Candidates: (Best fit distribution in marked in bold)

**Normal**	**Mean (SE)**	**Sd (SE)**	**Loglikelihood**	**AIC**	**BIC**
	**78.39 (0.75)**	**8.59 (0.53)**	**-471.2**	**946.3**	**952.1**

Gamma	**Shape (SE)**	**Rate (SE)**	**Loglikelihood**	**AIC**	**BIC**
	81.98 (10.07)	1.05 (0.13)	-471.7	947.4	953.1

Log normal	**LogMean**	**LogSd**	**Loglikelihood**	**AIC**	**BIC**
	4.35 (0)	0.11 (0)	-472.4	948.7	954.5

Logistic	**Location**	**Scale**	**Loglikelihood**	**AIC**	**BIC**
	78.45 (0.73)	4.83 (0.35)	-471.3	946.5	952.3

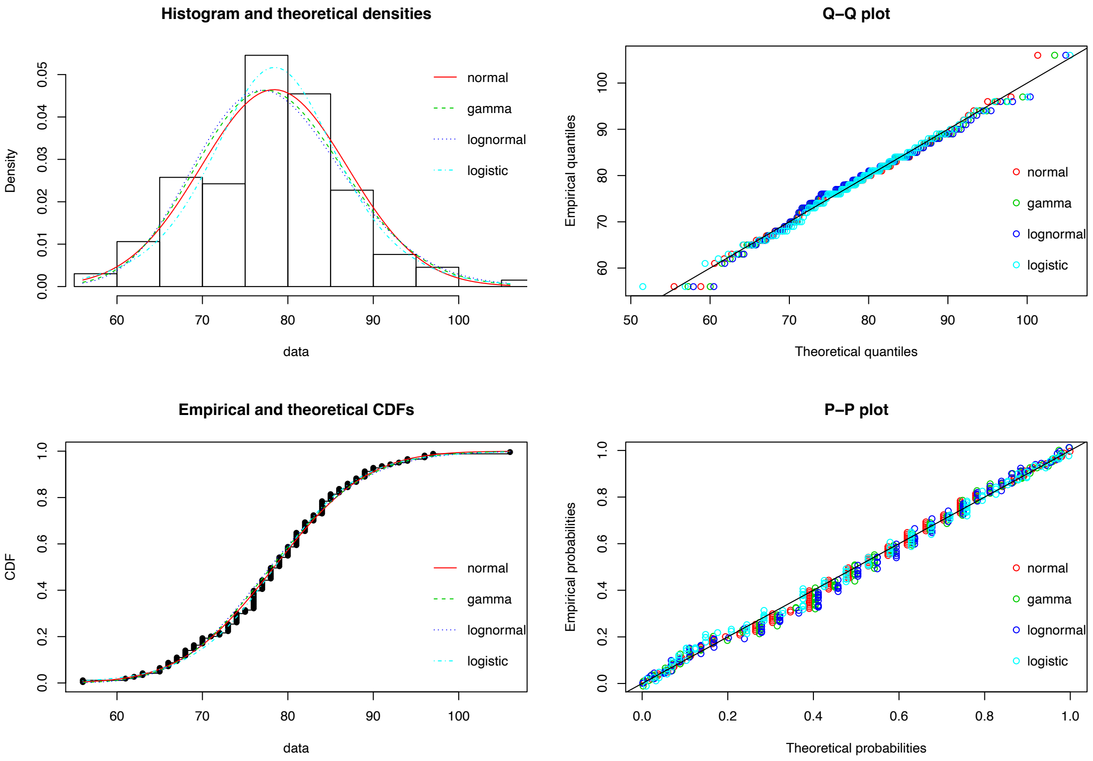

Albumin

Summary:

**min: 10 max: 34**	**median: 26**
**mean: 25.93**	**sample sd: 3.589**
**sample skewness: -1.849**	**sample kurtosis: 9.817**

Candidates: (Best fit distribution in marked in bold)

**Normal**	**Mean (SE)**	**Sd (SE)**	**Loglikelihood**	**AIC**	**BIC**
	**25.93 (0.31)**	**3.59 (0.23)**	**-356**	**716**	**721**

Gamma	**Shape (SE)**	**Rate (SE)**	**Loglikelihood**	**AIC**	**BIC**
	38.79 (4.75)	1.50 (0.18)	-374.5	752.9	758.7

Log normal	**LogMean**	**LogSd**	**Loglikelihood**	**AIC**	**BIC**
	3.24 (0.01)	0.18 (0.01)	-386	776.1	781.9

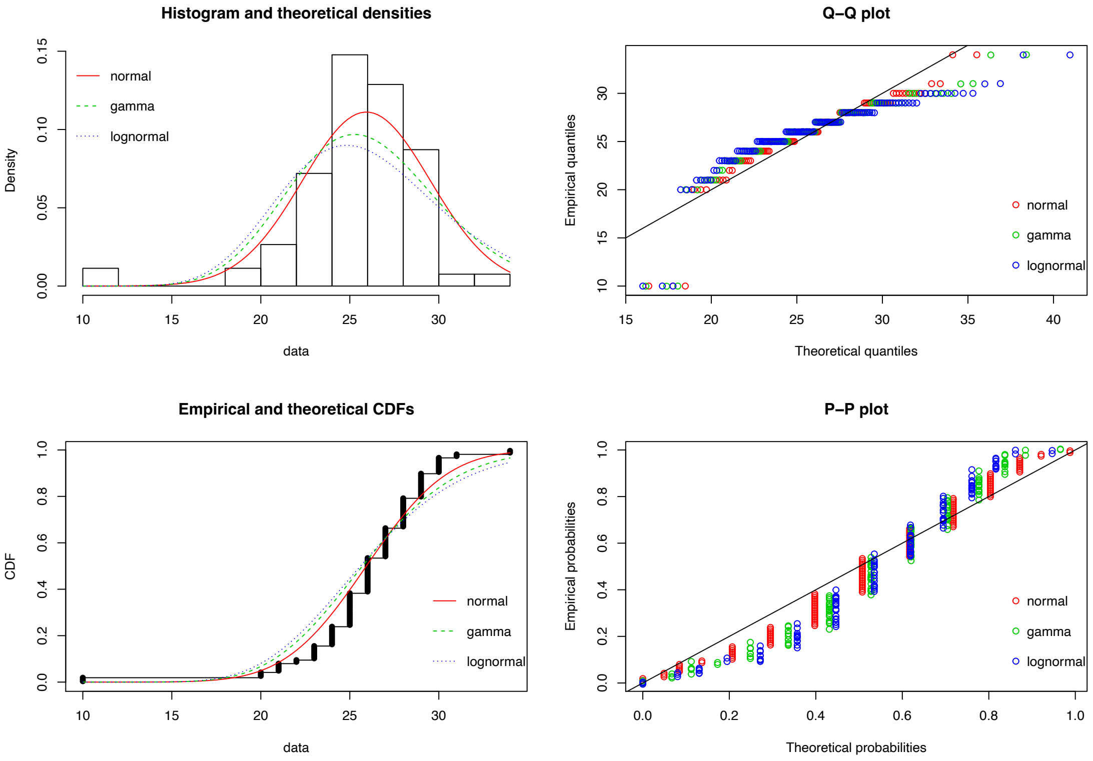

Globulin

Summary:

**min: 36 max: 100**	**median: 53**
**mean: 52.58**	**sample sd: 7.569**
**sample skewness: 1.665**	**sample kurtosis: 13.16**

Candidates: (Best fit distribution in marked in bold)

Normal	Mean (SE)	Sd (SE)	Loglikelihood	AIC	BIC
	52.58 (0.66)	7.57 (0.46)	-454.47	912.95	918.71

Gamma	Shape (SE)	Rate (SE)	Loglikelihood	AIC	BIC
	52.15 (6.40)	0.99 (0.12)	-448.49	900.99	906.76

**Log normal**	**LogMean**	**LogSd**	**Loglikelihood**	**AIC**	**BIC**
	**3.95 (0.01)**	**0.14 (0)**	**-446.96**	**897.92**	**903.68**

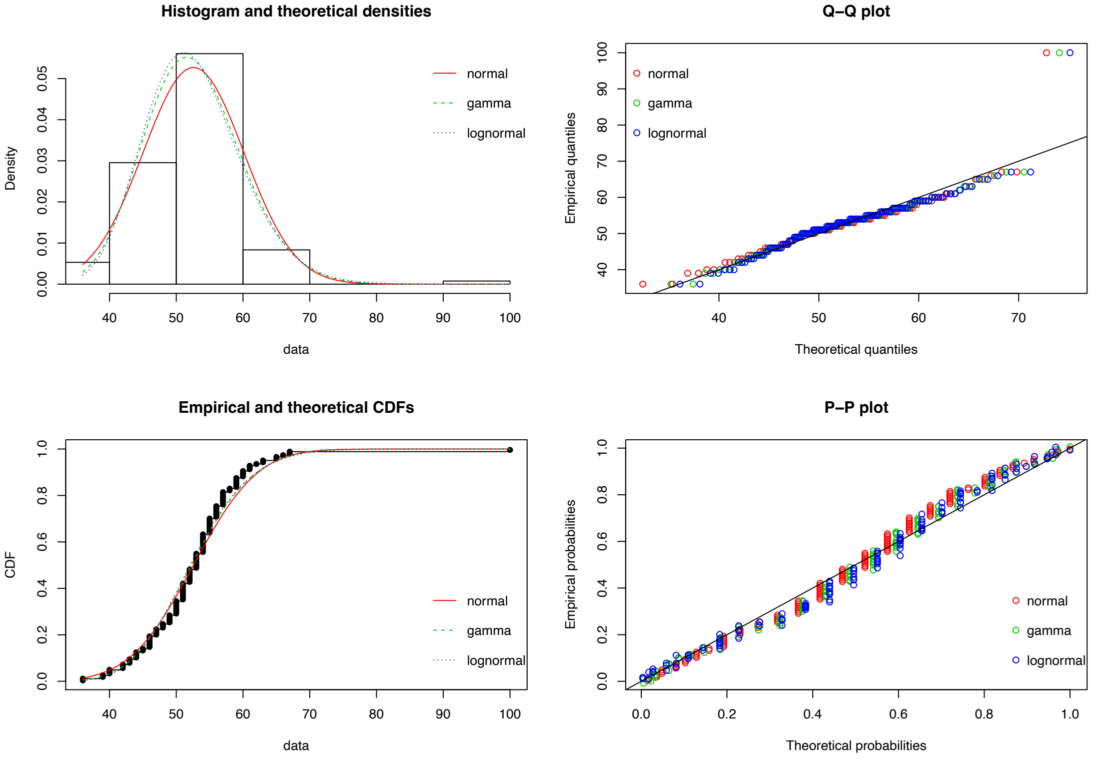

Uric acid

Summary:

**min: 0.1 max: 2.42**	**median: 0.55**
**mean: 0.6071**	**sample sd: 0.3212**
**sample skewness: 1.461**	**sample kurtosis: 7.55**

Candidates: (Best fit distribution in marked in bold)

	Mean (SE)	Sd (SE)	Loglikelihood	AIC	BIC
Normal	0.61 (0.02)	0.32 (0.02)	-55.5	115	121.6

	Shape (SE)	Rate (SE)	Loglikelihood	AIC	BIC
**Gamma**	**3.81 (0.37)**	**6.28 (0.65)**	**-30.81**	**65.63**	**72.18**

	LogMean	LogSd	Loglikelihood	AIC	BIC
Log normal	-0.63 (0.04)	0.54 (0.03)	-32.54	69.07	75.63

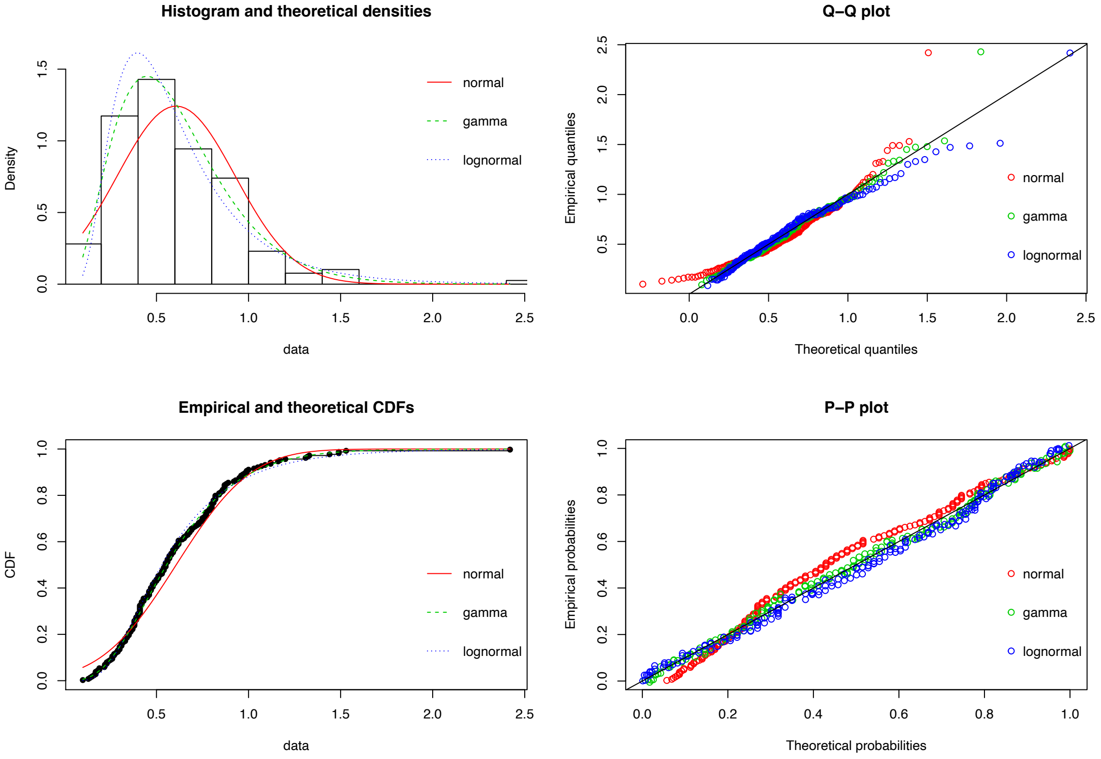

Sodium

Summary:

**min: 136 max: 172**	**median: 160**
**mean: 159.9**	**sample sd: 5.581**
**sample skewness: -0.6021**	**sample kurtosis: 4.726**

Candidates: (Best fit distribution in marked in bold)

**Normal**	**Mean (SE)**	**Sd (SE)**	**Loglikelihood**	**AIC**	**BIC**
	**159.88 (0.48)**	**5.58 (0.34)**	**-417.38**	**838.77**	**844.55**

Gamma	**Shape (SE)**	**Rate (SE)**	**Loglikelihood**	**AIC**	**BIC**
	807.32 (98.98)	5.05 (0.62)	-418.44	840.89	846.67

Log normal	**LogMean**	**LogSd**	**Loglikelihood**	**AIC**	**BIC**
	5.07 (0)	0.03 (0)	-419.04	842.08	847.86

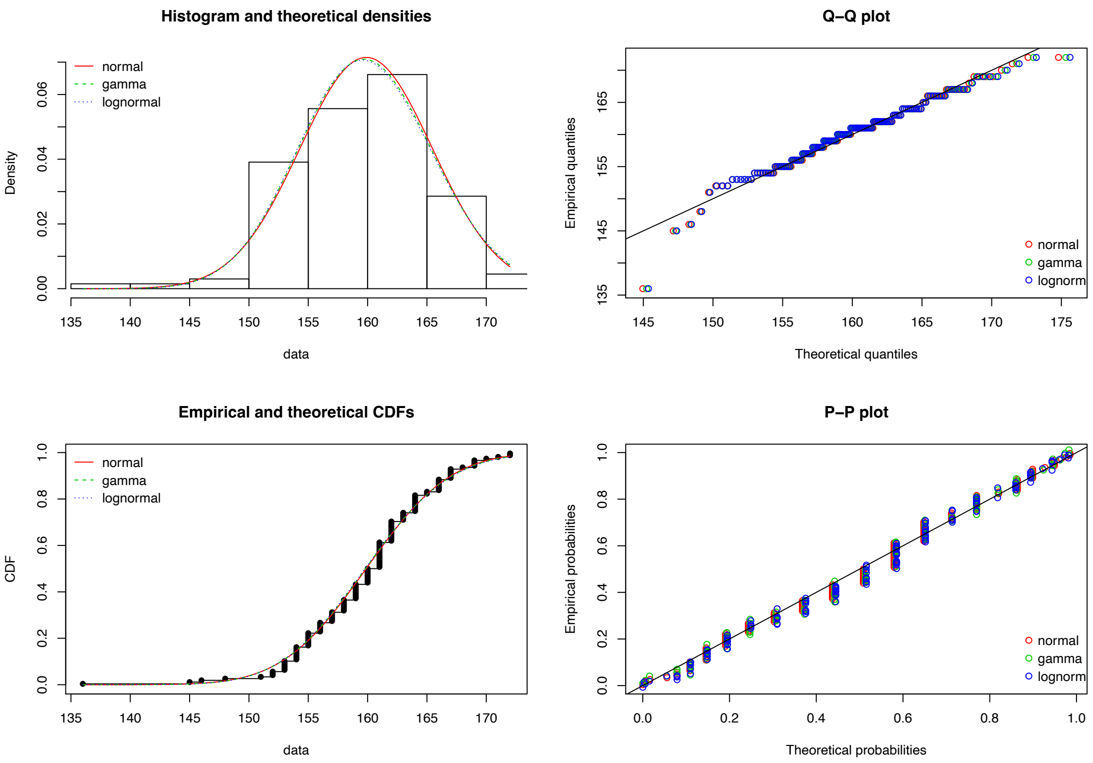

Potassium

Summary:

**min: 20.4 max: 58**	**median: 30.25**
**mean: 31.12**	**sample sd: 6.454**
**sample skewness: 1.721**	**sample kurtosis: 7.128**

Candidates: (Best fit distribution in marked in bold)

Normal	**Mean (SE)**	**Sd (SE)**	**Loglikelihood**	**AIC**	**BIC**
	31.12 (0.58)	6.45 (0.41)	-407.2	818.3	824

Gamma	**Shape (SE)**	**Rate (SE)**	**Loglikelihood**	**AIC**	**BIC**
	27.07 (3.42)	0.87 (0.11)	-396.2	796.4	802.1

**Log normal**	**LogMean**	**LogSd**	**Loglikelihood**	**AIC**	**BIC**
	**3.42 (0.02)**	**0.19 (0.01)**	**-392.1**	**788.2**	**793.9**

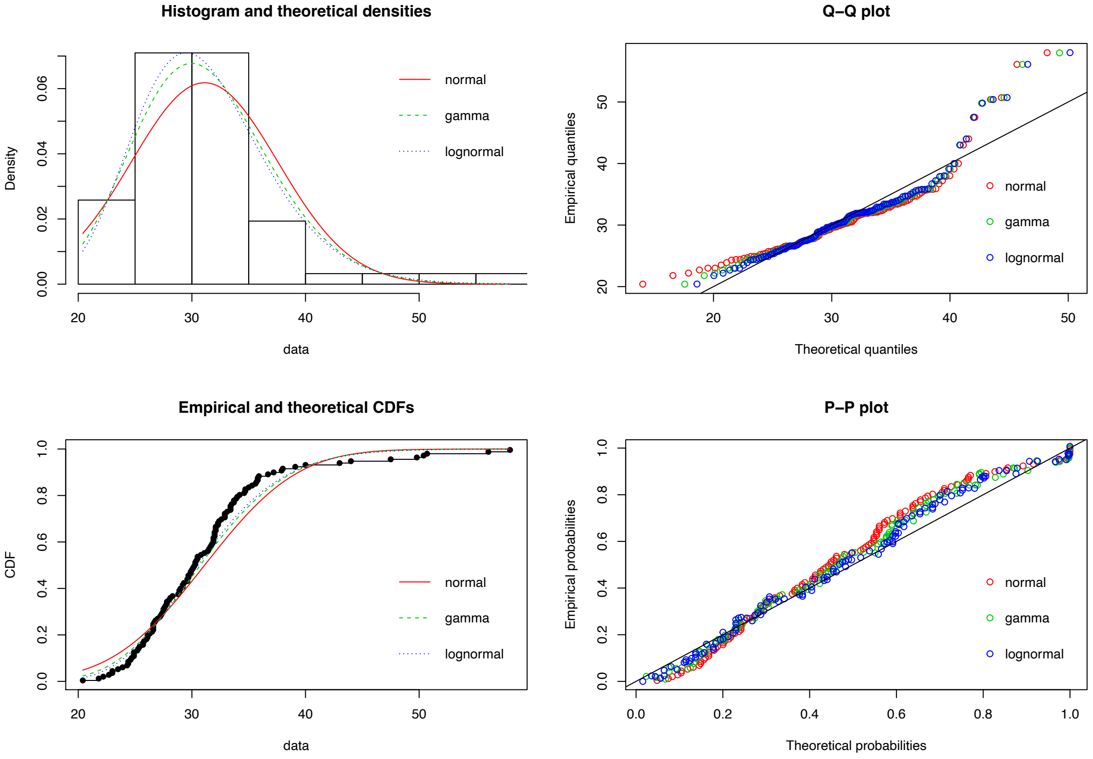

Chloride

Summary:

**min: 0.1 max: 2.42**	**median: 0.55**
**mean: 0.6071**	**sample sd: 0.3212**
**sample skewness: 1.461**	**sample kurtosis: 7.55**

Candidates: (Best fit distribution in marked in bold)

**Normal**	**Mean (SE)**	**Sd (SE)**	**Loglikelihood**	**AIC**	**BIC**
	**102.68 (0.51)**	**5.89 (0.36)**	**-424.5**	**853**	**858.8**

Gamma	**Shape (SE)**	**Rate (SE)**	**Loglikelihood**	**AIC**	**BIC**
	293.64 (35.99)	2.86 (0.35)	-426.7	857.4	863.2

Log normal	**LogMean**	**LogSd**	**Loglikelihood**	**AIC**	**BIC**
	4.63 (0)	0.06 (0)	-428	859.9	865.7

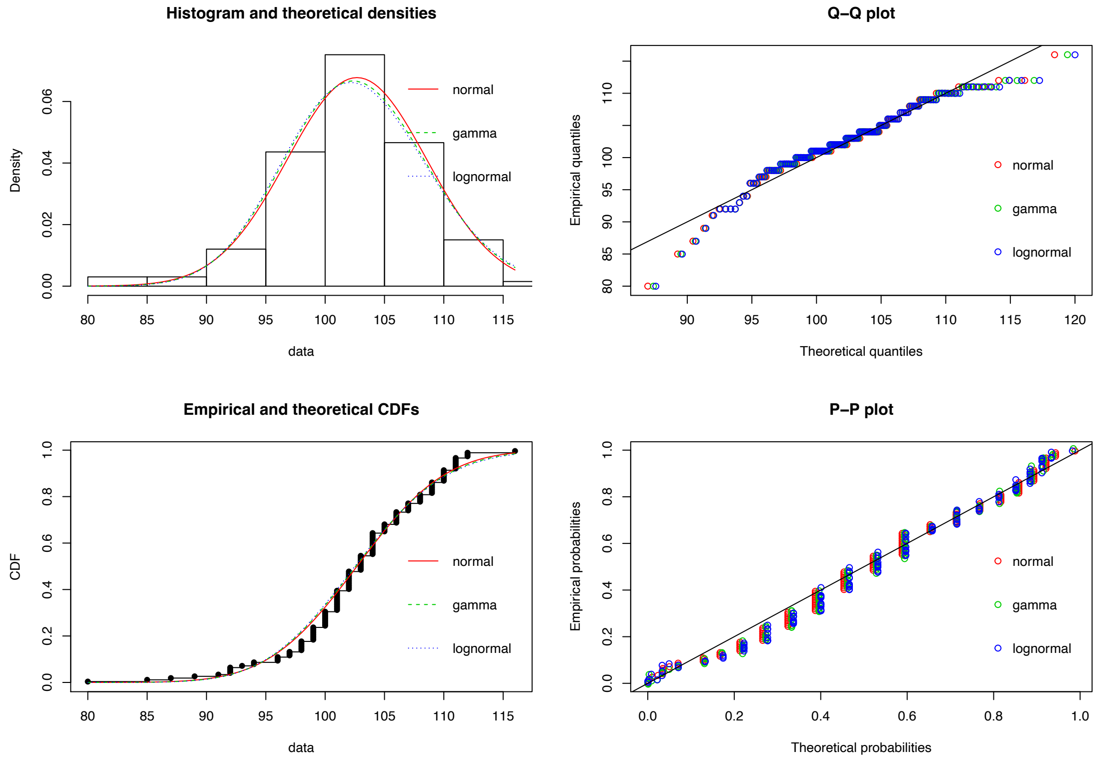

Fit of distribution for parasite richness by maximum likelihood estimation

Candidates: (Best fit distribution in marked in bold)

Poison	Lambda	S. Error	AIC	BIC
	**1.51**	**0.144**	**227**	**229**

Neg. binomial	Size (SE)	mu (SE)	AIC	BIC
	672.22 (4484)	1.51 (0.144)	229	234

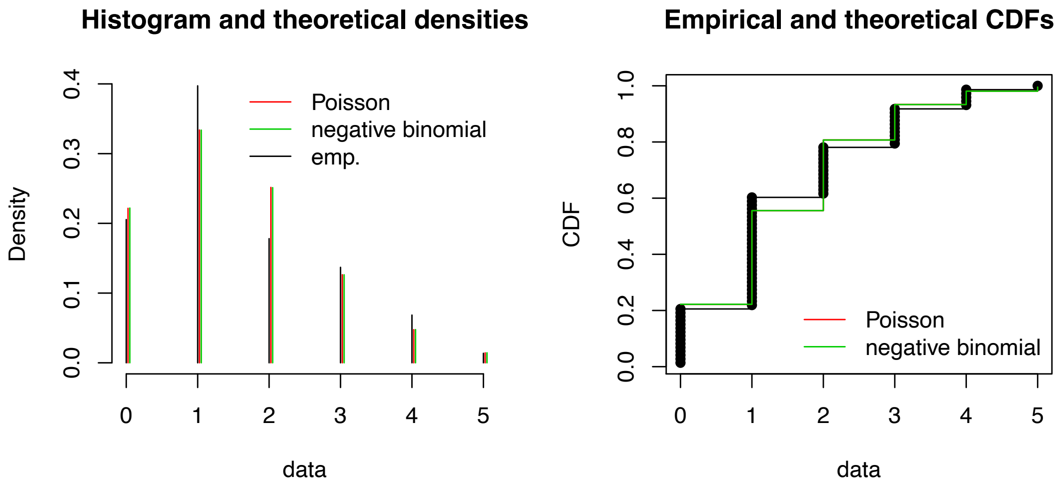

Fit of distribution for parasite prevalence by maximum likelihood estimation

Candidates: (Best fit distribution in marked in bold)

**Poison**	**Lambda**	**S. Error**	**AIC**	**BIC**
	**2.65**	**0.062**	3060	**3070**

Neg. binomial	**Size (SE)**	**mu (SE)**	**AIC**	**BIC**
	0.53 (0.04)	2.65 (0.15)	4532	4537

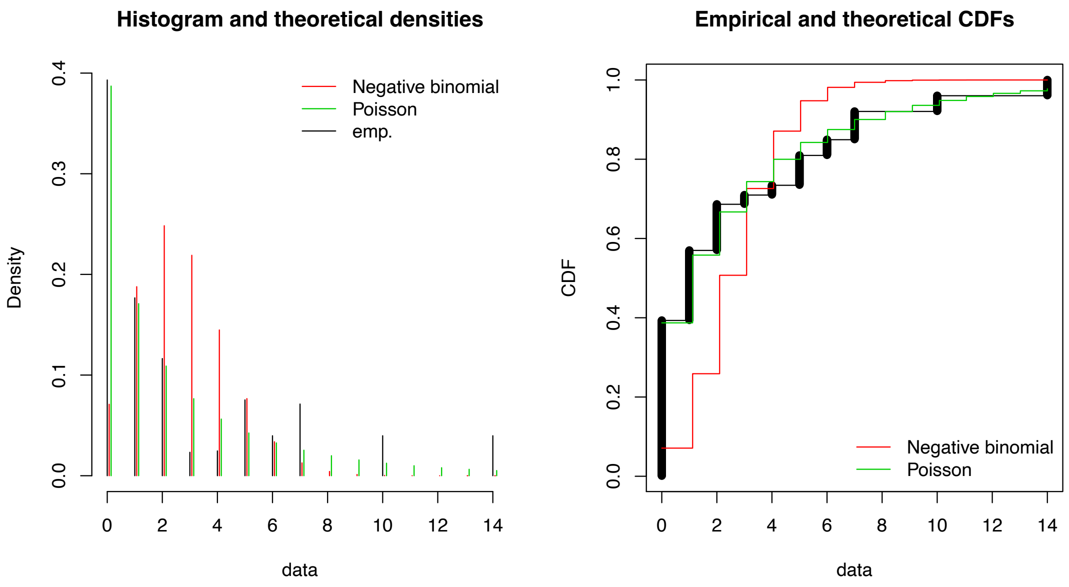

Supplement: Web_Material_coad055 [file web_material_coad055.zip › Supplement.pdf]
